# Heritable ER stress impairs mitochondrial metabolism and maintenance of hematopoietic stem cells after low-dose irradiation

**DOI:** 10.1016/j.isci.2026.114738

**Published:** 2026-01-19

**Authors:** Stephanie G. Moreno, Federica Ferri, Daniel Lewandowski, Vilma Barroca, Saiyiramii Devanand, Nathalie Dechamps, Paul-Henri Romeo, Nathalie Gault

**Affiliations:** 1 Commissariat à L’Energie Atomique et Aux Energies Alternatives (CEA), Université Paris Cité, Institut National de La Santé et de la Recherche Médicale (INSERM), Stabilité Génétique Cellules Souches et Radiations, 92260 Fontenay-Aux-Roses, France; 2 Université Paris-Saclay, INSERM, CEA, Stabilité Génétique Cellules Souches et Radiations, 92260 Fontenay-Aux-Roses, France; 3 Laboratoire Des Cellules Souches Hématopoïétiques et des Leucémies, Équipe Labellisée Ligue Contre Le Cancer, Équipe Niche Et Cancer Dans L’Hématopoïèse, Unité Mixte de Recherche (UMR) 1274 INSERM, CEA, 18 Route du Panorama, 92260 Fontenay-Aux-Roses, France; 4 Animal Experimentation Platform, DRCM, CEA, 92260 Fontenay-Aux-Roses, France; 5 Flow Cytometry Platform, DRCM, CEA, 92260 Fontenay-Aux-Roses, France

**Keywords:** Cell biology

## Abstract

How hematopoietic stem cells (HSCs) respond to low doses of radiation currently used in medicine is largely unknown. Here, we show that HSC exposed to a single 20 mGy dose of irradiation (20 mGy-HSC) exhibit, when proliferating, oxidative stress and altered metabolism associated with increased mitochondrial reactive oxygen species and mitochondrial Ca^2+^ overload. These mitochondrial defects arise from immediate and sustained endoplasmic reticulum (ER) stress, induced by proliferative 20 mGy-HSC through the activation of the eIF2α-ATF4 branch of the unfolded protein response (UPR). The ER stress is heritable and leads, in long-term quiescent 20 mGy-HSC, to the activation of the IRE1α-Xbp1 branch of UPR, which fails to restore ER homeostasis, resulting in a decreased long-term HSC pool. Finally, we show that this heritable ER stress leads to global DNA hypomethylation, partially reversed by the early inhibition of ER stress. Our studies illuminate how adaptive ER stress responses can lead to mitochondrial defects and HSC dysfunctions.

## Highlights

• Irradiated HSC at 20 mGy shows mitochondrial dysfunctions due to heritable ER stress
• Depending on the 20 mGy-HSC physiological state, different branches of UPR are activated
• The heritable ER stress of 20 mGy-HSC results in long-term HSC exhaustion
• ER stress leads to HSC DNA hypomethylation, partially reversed by its inhibition

## Graphical abstract

**Figure:**
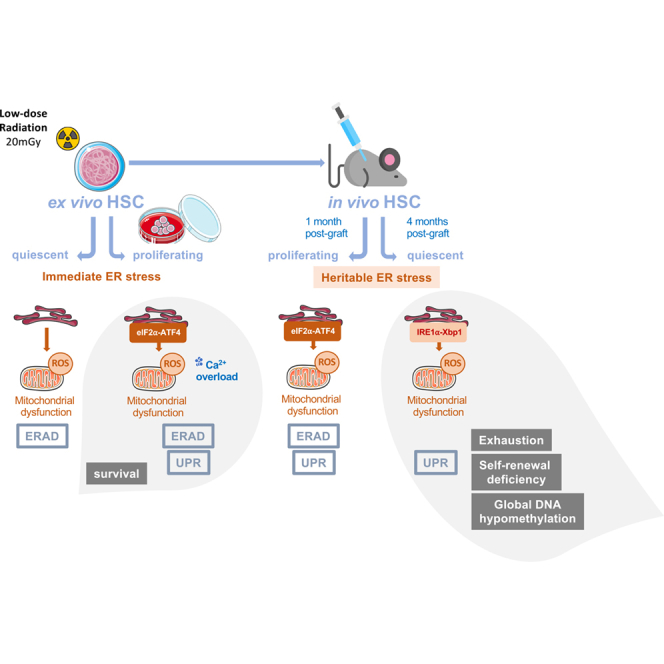

## Introduction

Hematopoiesis is highly sensitive to ionizing radiation (IR) and there is evidence that low doses of IR used in CT imaging and therapeutic applications may act as causative agents for cancers and non-cancer diseases such as cardiovascular disease.^1^ Indeed, an association between radiation exposure to doses lower than 50 mGy and risk of acute leukemia has been shown.^2^ In accordance, we and others have shown that, in contrast to multipotent progenitors (MPPs), hematopoietic stem cells (HSCs), and hematopoietic stem and progenitor cells (HSPCs) are hypersensitive to 20 mGy irradiation due to immediate oxidative stress.^3^^,^^4^ Murine HSC exposed to a single 20 mGy dose of irradiation (20 mGy-HSC) trigger specific cytoprotective pathways, including autophagy and the KEAP1-NRF2 signaling, but displayed persistent oxidative stress associated with phenotypic and functional alterations. These included myeloid-biased differentiation at the expense of lymphoid lineages, impaired homing, and reduced long-term contribution to hematopoiesis. Furthermore, using non-irradiated Rag2^−/−^γc^−/−^cKit^W/v^ recipient mice, transplantation of 20 mGy-HSC in competition with endogenous HSC showed a reduction in the number of irradiated HSC. This reduction was also observed after secondary transplantation in myeloablated recipient mice, indicating an altered long-term maintenance of these irradiated HSC.^3^ Interestingly, exposure of mice to a 20 mGy total body irradiation (TBI) under homeostatic conditions did not significantly affect HSC, indicating that the bone marrow microenvironment provides protection to HSC. However, when the same low-dose TBI was applied to cycling HSC, for example, under inflammatory conditions, a significant decrease in HSC was observed long-term post-TBI. These findings indicated that HSC radiosensitivity to 20 mGy TBI is dynamically influenced by their proliferative status.^3^ While these functional defects highlight the long-term effects of low-dose irradiation on HSC, the mechanisms underlying the persistent oxidative stress are not defined. Mitochondria and endoplasmic reticulum (ER) are organelles potentially involved in the effects of low doses of irradiation on HSC, as they are the major sources of reactive oxygen species (ROS)^5^ and are critical for HSC proliferation and metabolism. Under homeostasis, HSC are quiescent, exhibit low mitochondrial metabolism despite high mitochondrial content, and rely on anaerobic glycolysis and fatty acid oxidation for energy production. This state maintains low levels of ROS, preserving the integrity of the HSC pool.^6^ In response to stress, HSC can exit quiescence and proliferate to restore hematopoietic homeostasis. This transition is associated with a metabolic switch from cytoplasmic anaerobic glycolysis to increased mitochondrial metabolism, which activates oxidative phosphorylation (OXPHOS) to increase ATP production.^7^ This switch is accompanied with increased levels of ROS, which can lead to mitochondrial dysfunctions, DNA damages contributing to genomic instability and loss of HSC self-renewal.^8^ Mitochondria also regulate several processes in HSC such as their metabolism during commitment and differentiation,^9^ their epigenome,^10^ and their apoptosis.^9^ These findings indicate that mitochondria act as a central regulator of HSC functions and that maintaining proper mitochondrial homeostasis is crucial to prevent HSC dysfunction.^9^

Quiescent HSCs have a low level of protein synthesis that takes place in the ER. ER stress in HSC triggers ROS generation^11^ and occurs under physiological conditions, such as HSC expansion^12^ and under stress conditions such as inflammation^13^ or 5-fluorouracil treatment.^14^ Of note, reducing ER stress in HSC improves their self-renewal.^15^ In response to ER stress, HSC activate several adaptive mechanisms, i.e., enhanced ER-Associated protein Degradation (ERAD), autophagy, and unfolding protein response (UPR). Activation of these pathways depends on the intensity and duration of ER stress. ERAD is responsible for the clearance of misfolded proteins in the ER by targeting them for proteasomal degradation. ERAD is essential for maintaining HSC in a metabolically inactive and quiescent state with low levels of ROS^16^ and is crucial for regulating the interaction between HSC and their niche.^17^ Autophagy helps to degrade misfolded/unfolded or damaged proteins and aggregates, as well as specific organelles such as mitochondria or ER. Autophagy plays an important role in maintaining HSC self-renewal,^18^ in balancing myeloid-lymphoid differentiation^19^ and in preserving the regenerative potential of HSC both under physiological conditions and in response to metabolic stress.^20^ It protects HSC from cell death^21^ or premature aging by eliminating hyperactive mitochondria.^22^^,^^23^ UPR can be activated when misfolded or unfolded proteins accumulate in the ER, reducing the biogenesis of defective translation products, unfolded/misfolded, and aggregated proteins. In HSC, the UPR response to ER stress is highly dependent on their metabolism. Metabolic stress induced by amino acid deprivation in human HSC cultures induced an adaptive cytoprotective response by activating the PERK-ATF4 branch of UPR.^24^ Lipopolysaccharide-induced HSC hyperproliferation activates an adaptive ER stress response via the IREα-Xbp1 signaling pathway, promoting HSC survival and preserving their function.^13^ In contrast, prolonged ER stress in HSC can promote apoptosis via the IREα-Xbp1 signaling pathway of UPR, leading to HSC exhaustion.^25^ These results indicate an important role for ER in maintaining HSC function.

Mitochondria and ER are coordinated^26^ to control HSC fate, and calcium signaling closely connects the ER and mitochondria. When mitochondrial functions depend on calcium signaling, it is released from the ER into the cytosol and taken up by mitochondria, thereby regulating mitochondrial metabolism and/or morphology.^27^^,^^28^ Indeed, ER is a calcium reservoir, playing a crucial role in protein folding and processing. ER also functions as a signaling organelle where its calcium content is tightly regulated. In accordance, alterations in calcium levels, such as an ER reduction or a cytosolic increase, can disrupt ER proteostasis and lead to ER stress.^29^ HSCs are particularly sensitive to calcium signaling. Under homeostasis, calcium signaling and flux regulate the balance between “dormant” and “quiescent but active” HSC, dormant HSC exhibiting higher cytosolic calcium levels.^30^ In addition, a transient increase in intracellular calcium can initiate HSC divisions under stress conditions.^31^ During transplantation, HSC can sense and respond to fluctuations in their microenvironment calcium levels, with calcium signaling regulating their migration, homing, and localization in the bone marrow.^32^

In this study, we show that proliferative HSC previously irradiated at 20 mGy exhibited mitochondrial calcium overload, activated mitochondrial metabolism, and increased mitochondrial ROS compared to non-irradiated HSC. These metabolic changes are the result of an early and sustained ER stress in irradiated HSC. In proliferative irradiated HSC, ER stress activates the eIF2α-ATF4 branch of UPR, preventing apoptosis and autophagy. Quiescent HSC derived from transplanted irradiated HSC still exhibit ER stress that activates the IRE1α-Xbp1 branch of UPR, which fails to resolve oxidative stress and to maintain the HSC pool. In mice, this persistence of ER stress in HSC derived from transplanted irradiated HSC is associated with the global hypomethylation of genomic DNA, a hallmark of genomic instability. This study highlights how low-dose radiation of HSC induces a long-lasting ER stress that disrupts mitochondrial metabolism and modifies HSC epigenetic profile, thereby contributing to a gradual alteration in the maintenance and function of HSC.

## Results

### Increased oxidative stress in proliferative 20 mGy-irradiated hematopoietic stem cells associated with protein oxidative damage

After transplantation into lethally irradiated recipients, quiescent donor HSC undergo intensive proliferation to restore hematopoiesis. To investigate the impact of low-dose irradiation on this regenerative process, we transplanted CD45.2-conditioned recipient mice (9.5 Gy TBI leading to complete BM ablation) with CD45.1/CD45.2 donor LSK cells (Lin^neg^ Sca-1^+^ c-Kit^+^) previously exposed to 20 mGy or sham-irradiated (Figure S1A upper and lower). Bone marrow was harvested 15–30 days post-transplantation, i.e., during the early regenerative phase characterized by cycling HSC. Analysis of Ki-67 expression, DNA content, and intracellular ROS levels revealed that both 20 mGy- and sham-irradiated donor HSC similarly proliferated 15–30 days post-transplantation (Figure S1B, left and middle panels). However, proliferating HSC derived from 20 mGy-irradiated donor-LSK had an increased level of intracellular ROS compared to sham-irradiated controls (Figure S1B, right panel), indicating that low-dose irradiation did not impair proliferative capacity but altered the redox status of regenerating HSC.

**Figure 1:**
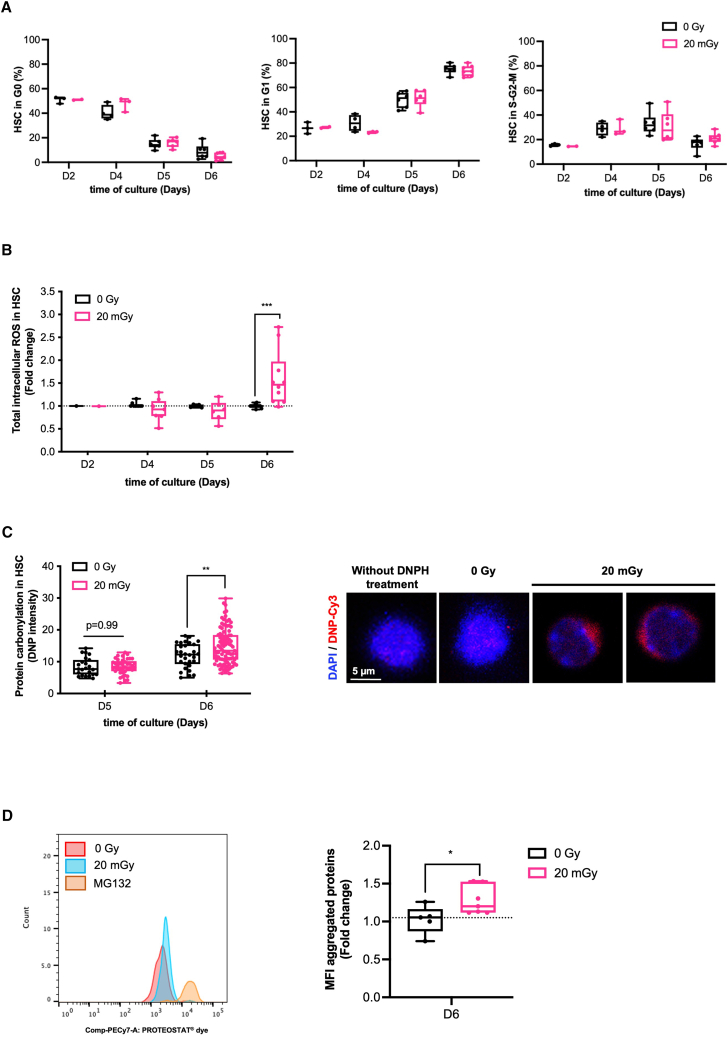
Increase in ROS occurs in proliferative irradiated HSC, causing protein oxidative damage (A) Kinetics of cell cycle in sham- (0Gy) and 20 mGy-irradiated (20 mGy) HSC at the indicated days of culture. Graphs show the percentage of HSC in G0 (left, Ki-67^neg^ Hoechst (Ho)^low^), in G1 (middle, Ki-67^pos^ Ho^low^), and in the S-G2-M phase of the cell cycle (right, Ki-67^pos^ Ho^hi^). *n* = 2 to 6 independent culture experiments. (B) Kinetics of total intracellular ROS in 0Gy and 20 mGy-HSC at the indicated days of culture. Graph shows total ROS levels in 20 mGy-HSC relative to 0Gy-HSC. *N* = 1 to 7 independent culture experiments. (C) Left, Quantification of protein carbonylation in 0Gy and 20 mGy-HSC at days 5 and 6 of culture, measured by 2,4-dinitrophenol (DNP). Right, Representative images show the overlay of DNA nuclear staining (DAPI, blue) and oxidized protein (DNP, red) at day 6 of culture. HSC without 2,4-dinitrophenylhydrazine (DNPH) treatment is shown as a negative control. Scale bar corresponds to 5 μm. (D) Detection of protein aggregates in 0Gy and 20 mGy-HSC at day 6 of culture. Left, representative plot of the Proteostat level staining. HSC treated with the proteasome inhibitor MG132 are used as a positive control. Right, the graph shows the fold change in 20 mGy-HSC over 0Gy-HSC. *N* = 5 to 7 independent culture experiments. Data are represented with a min to max box-and-whisker. Statistical significance was assessed using a Kruskal-Wallis test (A and B) or a two-sided Student’s t test (C) or a Wilcoxon-Mann-Whitney test (D). ∗*p* ≤ 0.05; ∗∗*p* ≤ 0.01; ∗∗∗*p* ≤ 0.001.

To mimic HSC entry into proliferation, HSCs (LSK FLK2^neg^ CD48 ^neg^) were isolated from bone marrow by cell sorting and cultured at low density in cytokine-supplemented medium (Figure S1C). After 6 days of culture, 80% of the cells retained the LSK FLK2 ^neg^ phenotype (Figure S1D) corresponding to a heterogeneous population containing both HSC (CD48 ^neg^) and early HSPC (CD48^+^) that are hypersensitive to low-dose irradiation,^3^ while the remaining 20% corresponded to more differentiated multipotent progenitors (MPP; LSK FLK2^+^) that are not hypersensitive to low-dose irradiation.^3^ Depending on the type of analyses, experiments were therefore performed using purified **HSC** or combined **HSC/HSPC** populations as indicated in each figure. On days 2 and 4 of culture, approximately 50% of the 20 mGy- and 0Gy-HSC were in G0. Then, 20 mGy- and 0Gy-HSC started to proliferate on day 5 of culture, and 95% of HSC were proliferating on day 6 of culture (Figure 1A). Similar levels of total ROS were detected in 20 mGy-HSC and 0Gy-HSC on days 2, 4, and 5, but the level of ROS significantly increased on day 6 only in 20 mGy-HSC (Figure 1B). The level of protein carbonylation, an irreversible oxidative modification, was used to assess the extent of oxidative stress in the 20 mGy-and 0Gy-HSC and showed increased protein oxidation in 20 mGy-HSC at day 6 of culture (Figure 1C). This increased level of protein oxidation was associated with higher protein aggregation in 20 mGy-HSC (Figures 1D and S1F). Taken together, these data show that proliferative 20 mGy-HSC exhibit oxidative stress with increased ROS level, which may lead to protein oxidation and aggregation.

### Increased levels of reactive oxygen species in 20 mGy irradiated hematopoietic stem cells are associated with increased mitochondrial metabolism

**Figure 2:**
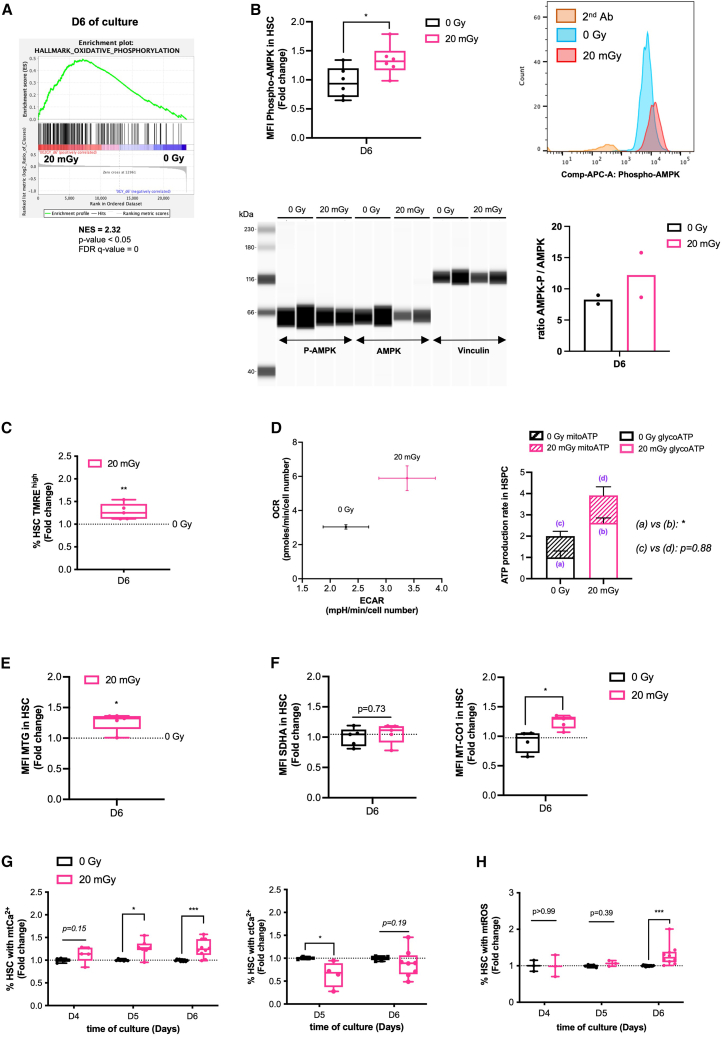
Increased levels of ROS in 20 mGy-HSC are associated with increased mitochondrial metabolism (A) Gene set enrichment analysis (GSEA) shows the oxidative phosphorylation signature positively correlated in the transcriptomic data of 20 mGy-HSC compared to 0Gy-HSC at day 6 of culture. FDR, false discovery rate; NES, normalized enrichment score; *n* = 3 independent culture experiments. (B) Intracellular phosphorylated AMPK (Thr172) levels by flow cytometry analysis (top) or detection in capillary western blot assay (WES) (Bottom) in 0Gy and 20 mGy-HSC at day 6 of culture. Top Left, the graph represents the mean fluorescence intensity (MFI) of P-AMPK in 20 mGy-HSC expressed as fold change relative to 0Gy-HSC. Top Right, representative FACS plot showing intracellular P-AMPK levels in 20 mGy-HSC and 0Gy-HSC. A negative control with only the secondary Ab was included to assess specificity. Bottom Left, WES assay for the detection of P-AMPK, AMPK, and Vinculin shown as gel-like view proteins in 0Gy-HSC and 20 mGy-HSC at day 6 of culture. Bottom right, Data are presented as P-AMPK to total AMPK ratio in 0Gy- and 20 mGy-HSC. *N* = 2 independent cell cultures. (C) Mitochondrial membrane potential using the tetramethylrhodamine ethyl ester (TMRE) probe at day 6 of culture. Data are expressed as the percentage of HSC with high TMRE intensity and relative to 0Gy-HSC. *N* = 5 independent cell cultures. (D) Quantification of ATP production from glycolysis (glycoATP) and OXPHOS (mitoATP) in 0Gy and 20 mGy-HSPC at day 6 of culture. Left, the graph shows the basal oxygen consumption rate (OCR, y axis) and the basal extracellular acidification rate (ECAR, x axis). Right, the graph shows the basal glycoATP and mitoATP normalized to cell number of 20 mGy-HSPC and 0Gy-HSPC; *n* = 4 independent cell cultures. (E) Mitochondrial mass using MitoTracker Green (MTG) labeling in 0Gy and 20 mGy-HSC at day 6 of culture. Graph represents the mean fluorescence intensity (MFI) in 20 mGy-HSC relative to 0Gy-HSC; *n* = 5 independent cell cultures. (F) Quantification of mitochondrial biogenesis in 0Gy and 20 mGy-HSC at day 6 of culture. Graph represents the MFI of SDHA (left) and MTCO1 (right) proteins in 20 mGy-HSC relative to 0Gy-HSC. *n* = 5 independent cell cultures. (G) kinetics of mitochondrial (mtCa^2+^) and cytosolic (ctCa^2+^) calcium in 0Gy and 20 mGy-HSC at the indicated days of culture. Left, graph represents the percentage of 20 mGy-HSC with mtCa^2+^ relative to 0Gy-HSC. Right, the graph represents the percentage of 20 mGy-HSC with ctCa^2+^ relative to 0Gy-HSC. n = 3–10 independent cell cultures. (H) Kinetics of mitochondrial ROS (mtROS) in 0Gy and 20 mGy-HSC at the indicated days of culture. Graph represents the percentage of 20 mGy-HSC with mtROS relative to 0Gy-HSC; n = 3–9 independent cell cultures. Data are represented with mean ± SEM or min to max box-and-whisker. Statistical significance was assessed using a Wilcoxon-Mann-Whitney test (B, C, D, E, and F) or a Kruskal-Wallis test (G and H). ∗*p* ≤ 0.05; ∗∗*p* ≤ 0.01; ∗∗∗*p* ≤ 0.001.

To determine the biological pathways by which 20 mGy irradiation enhances intracellular ROS in proliferative HSC, we performed a transcriptome analysis at day 6 of culture (Figure S2A). Purified 20 mGy- and 0Gy-HSC were compared by using the gene set enrichment analysis (GSEA), and the most changed genes for both groups are shown in Figure S2B. GSEA hallmark pathways analysis showed that, compared to 20 mGy-HSC, 0Gy-HSC were significantly enriched for genes involved in genomic stability during cell proliferation (G2/M checkpoint, E2F targets, and mitotic spindle) (Figure S2C). In contrast, enrichment of genes regulating oxidative phosphorylation (Figure 2A) and xenobiotic metabolism (Figure S2D) was significantly found in 20 mGy-HSC, suggesting an energetic stress with a metabolic switch from glycolysis to OXPHOS. AMPK is a key sensor of energetic stress; we thus studied its activation through Thr172 phosphorylation and found, at day 6 of culture, an increased phosphorylation of AMPK (P-AMPK) in 20 mGy-HSC by flow cytometry (Figure 2B upper left and right) or by WES analysis showing a higher P-AMPK/AMPK ratio in 20 mGy-HSPC compared to 0Gy-HSPC sorted by cell sorter after 6 days of culture (Figure 2B lower left and right). As AMPK activation is known to enhance OXPHOS and boost mitochondrial activity, we characterized mitochondrial function in proliferative HSC at day 6 of culture. We measured the mitochondrial transmembrane potential (MMP) and found an increased percentage of 20 mGy-HSC with a high MMP (Figure 2C; Figure S2E). This high MMP was associated with an increase in basal mitochondrial energetic metabolism in the 20 mGy-HSPC-enriched fraction as reflected by OCR and ECAR rates (Figure 2D left). Notably, Seahorse analysis was performed on the HSPC fraction (more than 80% of the cells after 6 days of culture in both 20 mGy and 0Gy conditions) rather than on the sorted HSC fraction, as cell sorting suppressed HSPC adhesion. ATP production via glycolysis versus OXPHOS was quantified from these OCR and ECAR traces (Figure S2F left and right) using the Agilent Seahorse XF Real-Time ATP Rate Assay Report Generator. Compared to 0Gy-HSPC, the rate of ATP production was higher in 20 mGy-HSPC with a 3-fold increase in glycolytic ATP production, while ATP production by OXPHOS remained similar to that of the 0Gy-HSPC (Figure 2D right). We then examined mitochondrial mass and found that it was 1.3-fold higher in 20 mGy-HSC compared to 0Gy-HSC at day 6 of culture (Figure 2E). This increase in mass was associated with an increased level of the mitochondrial DNA-encoded mitochondrial protein MTCO1 without any change in the level of nuclear DNA-encoded mitochondrial protein SDHA (Figure 2F), consistent with an increase in mitochondrial content. Since calcium (Ca^2+^) influx into mitochondria is required for mitochondrial biogenesis and metabolism activation, we quantified cytosolic (ct) and mitochondrial (mt) Ca^2+^ levels in 20 mGy- and 0Gy-HSC on days 4, 5, and 6. On days 5 and 6 of culture, more than 20 mGy-HSC contained significantly elevated levels of mtCa^2+^ (Figure 2G, left). This increased percentage of 20 mGy-HSC with high levels of mtCa^2+^ was associated with a decreased percentage of 20 mGy-HSC with decreased levels of ctCa^2+^ on day 5 but not on day 6 (Figure 2G, right). In accordance with Ca^2+^ and ROS cross-talk,^33^ we found an increased percentage of 20 mGy-HSC with high mtROS at day 6 of culture (Figure 2H). Taken together, these results show that proliferative 20 mGy-HSC exhibit an energetic stress that enhances metabolism with mitochondrial Ca^2+^ overload and mitochondrial ROS overproduction.

### 20 mGy irradiation induces an immediate and persistent endoplasmic reticulum stress adapted by quiescent hematopoietic stem cells through endoplasmic reticulum-associated protein degradation activation

**Figure 3:**
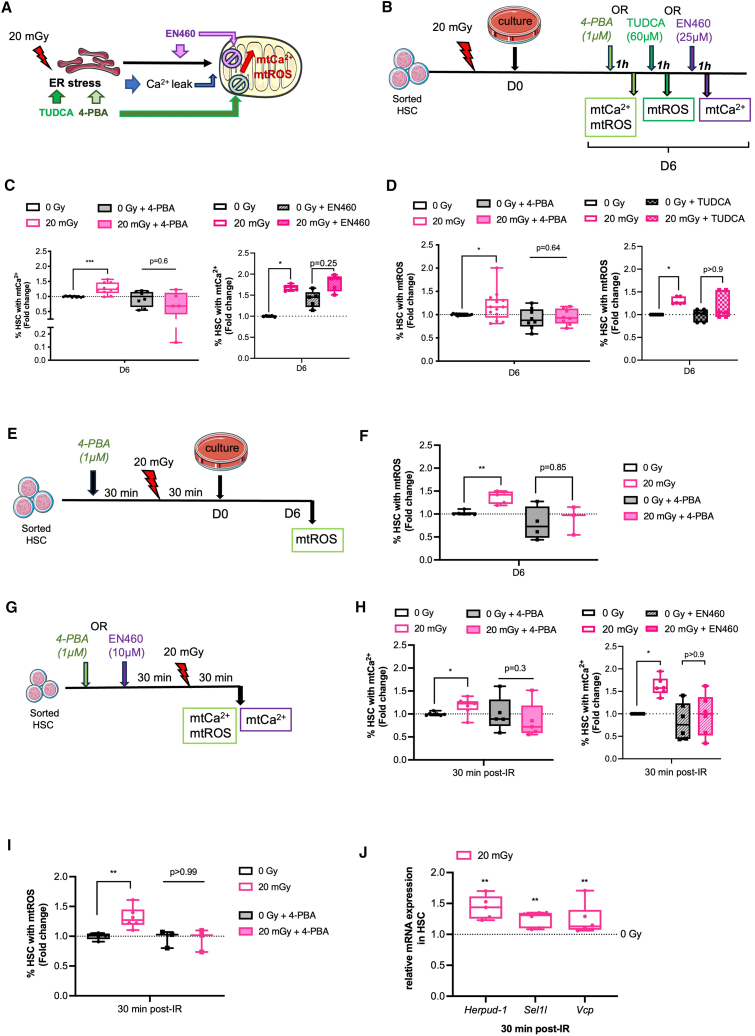
20mGy irradiation induces an immediate and persistent ER stress adapted by quiescent HSC through ERAD activation (A) Schematic representation of ER stress, which contributes to mitochondrial calcium overload and mitochondrial ROS generation. The selective ERO1a inhibitor EN460 was used to test the role of ERO1α in ER-to-mitochondria calcium flux, while 4-PBA and TUDCA assessed the general effects of ER stress on mitochondria. (B) Scheme of the experimental design for 4-PBA, TUDCA, or EN460 treatment at day 6 of culture. 0Gy and 20 mGy-HSC were cultured for 6 days and treated or not with inhibitors 1 h prior the assessment of mtCa^2+^ and mtROS in HSC. (C) Graph shows the percentage of 20 mGy-HSC with mtCa^2+^ levels relative to 0Gy-HSC treated or not with 4-PBA or EN460; n = 5–10 independent cell cultures. (D) Graph represents the percentage of 20 mGy-HSC with mtROS levels relative to 0Gy-HSC treated or not with 4-PBA or TUDCA; n = 5–10 independent cell cultures. (E) Scheme of the experimental design for 4-PBA treatment before irradiation. Sorted HSC were treated or not with 4-PBA 30 min prior to 0Gy or 20 mGy irradiation for a 1-h total duration before culture. mtROS levels in HSC were assessed after 6 days of culture. (F) Graph shows the percentage of 20 mGy-HSC with mtROS levels relative to 0Gy-HSC treated or not with 4-PBA. n = 3–5 independent cell cultures. (G) Scheme of the experimental design for 4-PBA or EN460 treatment 30 min before irradiation and assessment of mtCa^2+^ and mtROS 30min after irradiation. (H) Graph represents the percentage of 20 mGy-HSC with mtCa^2+^ levels relative to 0Gy-HSC treated or not with 4-PBA or EN460. *N* = 8 independent cell cultures. (I) Graph represents the percentage 20 mGy-HSC with mtROS compared to 0Gy-HSC. n = 3–5 independent cell cultures. (J) mRNA levels of ERAD genes (*Herpud-1, Sel1l, and Vcp)* in 20 mGy-HSC relative to 0Gy-HSC. *n* = 5 independent cultures. Data are represented with min to max box-and-whisker. Statistical significance was assessed using a Kruskal-Wallis (C, D, F, H, and I) or a Wilcoxon-Mann-Whitney (J). ∗*p* ≤ 0.05; ∗∗*p* ≤ 0.01; ∗∗∗*p* ≤ 0.001.

To investigate whether mitochondrial ROS in 20 mGy-HSC is a consequence of ER stress, which stimulates Ca^2+^ transfer from the ER to the mitochondria,^34^^,^^35^ 20 mGy- and 0Gy-HSC were treated with 4-phenylbutyric acid (4-PBA), a known ER stress inhibitor. One-hour treatment with 4-PBA on day 6 of culture (Figures 3A and 3B) reduced both the percentage of 20 mGy-HSC with high mtCa^2+^ (Figure 3C left) and high mtROS (Figure 3D left) to the levels observed in 0Gy-HSC. To confirm that the oxidative stress was indeed dependent on ER stress, we treated HSC with a second ER stress inhibitor, Tauroursodeoxycholic acid (TUDCA),^15^ which has a distinct mechanism of action. One hour treatment with TUDCA on day 6 decreased the percentage of 20 mGy-HSC with high mtROS to levels comparable to 0Gy-HSC (Figure 3D, right). We next investigated the role of ERO1α in mediating ER-to-mitochondria Ca^2+^ transfer. Treatment of 20 mGy-HSC with the ERO1α inhibitor EN460^36^ for 1 h on day 6 (Figures 3A and 3B) normalized the percentage of cells with high mtCa^2+^ to that of 0 Gy controls (Figure 3C, right), indicating that ERO1α mediates this calcium flux. To explore whether ER stress occurs immediately after irradiation and contributes to early mitochondrial responses, HSC were treated with 4-PBA for 1 h prior to 20 mGy irradiation (Figure 3E). Pre-treatment with 4-PBA prevented the increase in the percentage of HSC with high mtROS after 6 days of culture (Figure 3F). Moreover, measurements performed 30 min post-irradiation (Figure 3G**)** revealed that 20 mGy-HSC exhibited elevated mtCa^2+^ (Figure 3H) and mtROS levels (Figure 3I), which were restored to baseline by 4-PBA pre-treatment. Finally, to determine whether ERO1α also mediates the immediate mitochondrial response, HSC were pre-treated with EN460 prior to irradiation (Figure 3G**)**. This treatment completely prevented the irradiation-induced increase in mtCa^2+^ (Figure 3H, right), demonstrating that ERO1α-mediated calcium flux is critical both immediately after irradiation and at day 6 of culture. These results indicate that mtROS accumulation in 20 mGy HSC is driven by ER and suggest that ER stress occurred immediately after irradiation and persisted until day 6 of culture.

Cells adapt to ER stress by activating autophagy or/and quality control machinery, including ERAD or UPR, which consists of a central sensor of ER stress, the Binding immunoglobulin Protein (BiP), also known as HSPA5, and three downstream activating branches (IREα-Xbp1, eIF2α-ATF4, and ATF6) (Figure S3A). We have previously shown that 20 mGy-HSC activates autophagy immediately after irradiation.^3^ No changes in *Hspa5*/*Bip* mRNA expression and in intracellular BiP protein expression were found between 20 mGy-HSC and 0Gy-HSC (Figures S3B and S3C). As the IREα-Xbp1 branch is the major UPR branch activated in HSC following various ER stresses,^37^ we investigated IRE1α-triggered *Xbp1* splicing (sp*Xbp1*) and increased *Xbp1*mRNA level regulated by sp*Xbp1* itself or the activated ATF6 UPR branch. We did not find any increased *Xbp1* splicing (Figure S3D), or any increased *Xbp1* mRNA level 30 min after 20 mGy irradiation of HSC (Figure S3E), indicating that canonical UPR activation does not occur in quiescent HSC immediately after irradiation. In contrast, 20 mGy-HSC showed increased mRNA levels of several ERAD genes, such as *Herpud-1*, *Sel1l,* and *Vcp* 30 min after irradiation (Figure 3J). Taken together, these results indicate that low-dose irradiation induces an immediate ER stress in quiescent HSC, leading to mitochondrial Ca^2+^ overload and overproduction of mtROS and triggering an early adaptive response through ERAD and autophagy activation.^3^ This stress persists in proliferating HSC, as shown by continued mitochondrial alterations at day 6, which are prevented by treatment with several ER stress inhibitors.

### Endoplasmic reticulum stress in proliferative 20 mGy-HSC triggered the activation of the eIF2α-ATF4 axis of unfolded protein response and promotes hematopoietic stem cell survival

**Figure 4:**
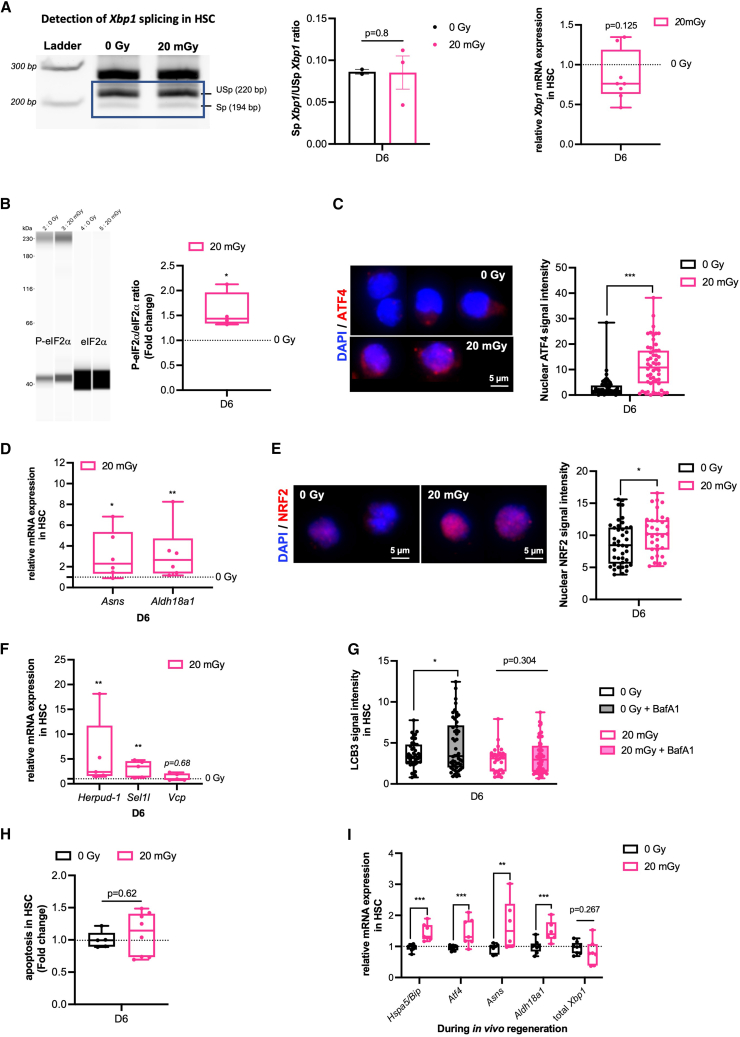
ER Stress in proliferative 20mGy-HSC triggered activation of the eIF2α-ATF4 axis of UPR and promotes HSC survival (A) Left, Splicing of *Xbp1* mRNA in 0Gy-HSC and 20 mGy-HSC at day 6 of culture. The sizes of the spliced (Sp) and the unspliced (USp) forms are indicated. Middle, Graph represents the ratio of Sp to USp *Xbp1* mRNA. Right, the graph shows total *Xbp1* mRNA levels in 20 mGy-HSC relative to 0Gy-HSC. n = 3–7 independent cell cultures. (B) Left, representative simple Western assay of the phosphorylated and total eIF2α proteins in 0Gy-HSC and 20 mGy-HSC at day 6 of culture. Right, Data are presented as the ratio of phosphorylated eIF2α (P-eIF2α) to total eIF2α and relative to 0Gy-HSC. *n* = 4 independent cell cultures. (C) Left, representative images from 0 Gy to 20 mGy-HSC at day 6 of culture showing ATF4 (red) localization. DAPI (blue) was used as a nuclear counterstain. Scale bar represents 5 μm. Right, the graph shows nuclear ATF4 signal intensity. A minimum of 30 cells/condition was analyzed. (D) mRNA levels of ATF4 target genes (*Asns, Aldh18a1)* in 20 mGy-HSC relative to 0Gy at day 6 of culture. *n* = 6 independent cell cultures. (E) Left, Representative images from 0 Gy to 20 mGy-HSC at day 6 of culture showing NRF2 (red) nuclear translocation. DAPI (blue) was used as a nuclear counterstain. Scale bar represents 5 μm. A minimum of 30 cells was quantified. Right, the graph represents the nuclear NRF2 signal intensity. (F) mRNA levels of ERAD genes (*Herpud-1, Sel1l and Vcp)* in 20 mGy-HSC relative to 0Gy-HSC at day 6 of culture. *n* = 5 independent cultures. (G) Autophagy measurement performed in the presence or absence of bafilomycin A1 (BafA1) by the quantification of the intracellular fluorescence of puncta-LC3B in 0 mGy-HSC and 20 mGy-HSC at day 6 of culture. A minimum of 30 cells per condition was analyzed. (H) Apoptosis measurement, via cleaved caspase 3/7 analysis in 0Gy and 20 mGy-HSC at day 6 of culture. Data are presented relative to 0Gy. *n* = 7 independent cell cultures. (I) Relative mRNA levels of indicated genes related to UPR in proliferating HSC from mice transplanted with 0Gy or 20 mGy-LSK. Data are presented as fold change relative to 0Gy-HSC. *n* = 7. Data are represented with min to max box-and-whisker. Statistical significance was assessed using a Wilcoxon-Mann-Whitney (A, B, D, F, H, and I) or a Two-sided Student’s t test (C, E, and G). ∗*p* ≤ 0.05; ∗∗*p* ≤ 0.01; ∗∗∗*p* ≤ 0.001.

Proliferation in HSC requires increased protein synthesis, which can induce ER stress and activate the adaptive survival signaling via the IRE1α-XBP1 pathway.^13^ As we observed ER stress in 20 mGy HSC, we investigated UPR signaling after 6 days of culture (Figure S4A). Compared to proliferative 0Gy-HSC, proliferative 20 mGy-HSC did not show increased *Xbp1* splicing (Figure 4A, left and middle) nor *Xbp1* mRNA level (Figure 4A, right). In contrast, we observed a 1.4-fold increase in eIF2α phosphorylation in 20 mGy-HSC compared to 0Gy-HSC (Figure 4B). The increase in eIF2α phosphorylation in 20 mGy-HSC was associated with increased ATF4 protein expression and its nuclear translocation (Figure 4C) and increased mRNA expression of ATF4 target genes such as *Asns* and *Aldh18a1* (Figure 4D). Activation of the eIF2α-ATF4 branch can promote NRF2 nuclear translocation, ERAD activation, autophagy, or apoptosis (Figure S3A). Immunofluorescence also showed the increased NRF2 nuclear translocation in 20 mGy-HSC (Figure 4E). The ERAD pathway was activated as shown by increased mRNA levels of the ERAD-related genes *Herpud-1* and *Sel1l* (Figure 4F). Autophagy was assessed by quantifying LC3B levels in 20 mGy-HSC and 0Gy-HSC in the presence or absence of an autophagy inhibitor, bafilomycin A1 (BafA1). We observed similar fluorescence intensities of cytoplasmic LC3B in 20 mGy-HSC and 0Gy-HSC in the absence of BafA1, and the intensity level was not further increased by the addition of BafA1 in 20 mGy-HSC, in contrast to 0Gy-HSC (Figure 4G), suggesting low autophagy activity in 20 mGy-HSC. Finally, we investigated whether the eIF2α-ATF4 branch of UPR induces apoptosis. Apoptosis was not increased in 20 mGy-HSC compared to 0Gy-HSC (Figure 4H). Activation of the eIF2α-ATF4 branch of the UPR in proliferative 20 mGy-HSC was also examined *in vivo* shortly after transplantation into conditioned mice. 20 mGy- and 0Gy-HSC were purified during the hematopoietic regenerative phase, and mRNA levels of the ER chaperone *HSPA5*/*BiP*, *ATF4,* and ATF4-target genes, as well as *Xbp1,* were quantified (Figure S4B). We found increased mRNA levels of *Hspa5/BiP*, *Atf4,* and the ATF4-target genes *Asns* and *Aldh18a1*, but there was no increase in *Xbp1* mRNA expression, suggesting eIF2α-ATF4 activation of UPR *in vivo* during the proliferation of 20 mGy-HSC (Figure 4I).

Taken together, these data indicate that ER stress is associated with eIF2α phosphorylation and nuclear increase expression of ATF4 in proliferative 20 mGy-HSC without enhancing autophagy or apoptosis, allowing HSC to adapt to ROS induced by ER stress.

### Low dose irradiation triggers a persistent endoplasmic reticulum stress-induced reactive oxygen species, leading to impaired long-term maintenance of hematopoietic stem cells

To study the impact of ER stress on the clonogenic potential of 20 mGy-HSC *in vitro*, HSC were cultured for 6 days, purified by cell sorting, and plated in methylcellulose for CFU assay (Figure S5A). Both 20 mGy-HSC and 0Gy-HSC produced the same number of primary colonies (Figure S5B, left panel). However, in secondary plating, 20 mGy-HSC produced fewer colonies than 0Gy-HSC, and this reduction was prevented by 4-PBA treatment (Figure S5B, right panel). These results indicate that ER stress reduces the clonogenic potential of HSC.

**Figure 5:**
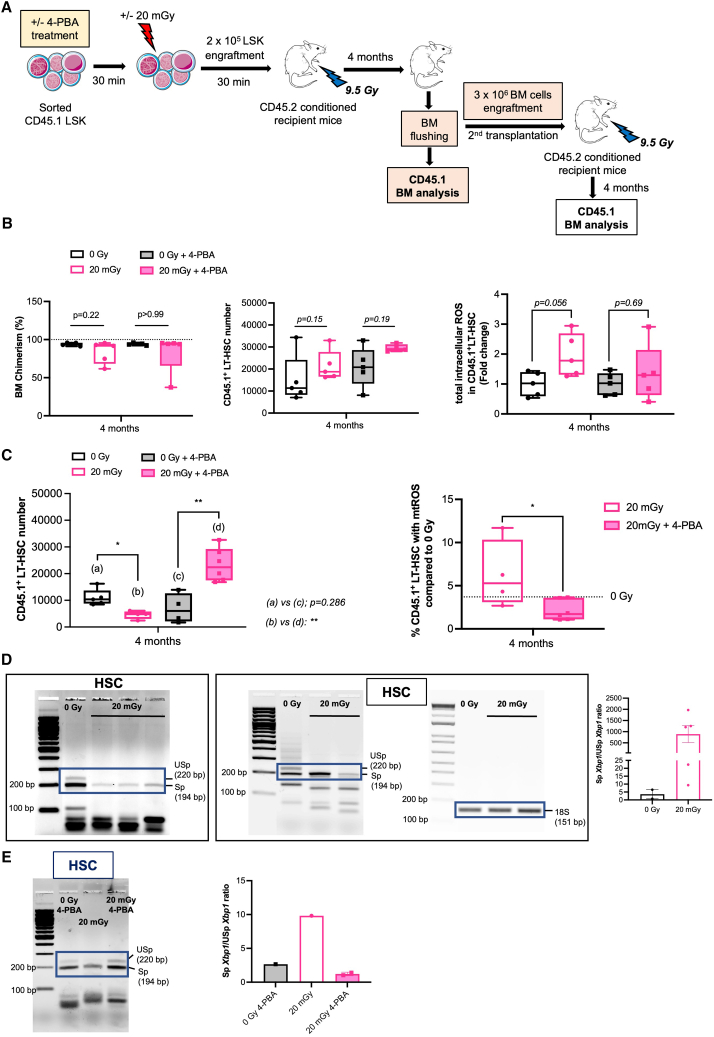
Low dose irradiation triggers a persistent ER stress-induced ROS, leading to the exhaustion of HSC (A) Experimental design for serial transplantation experiments. First transplantations were performed with 20,000 CD45.1+ LSK irradiated at 20 mGy or sham-irradiated, pretreated or not with 4-PBA, and transplanted into CD45.2+ conditioned recipient mice (9.5 Gy-TBI). The 4-PBA treatment lasts for 1 h, starting 30 min before irradiation. Secondary transplantations were performed using 3x10^6^ CD45.1+ whole BM cells, harvested from the primary recipient mice and transplanted into CD45.2+ conditioned recipient mice. Bone marrow was analyzed at the indicated time points. (B) Bone marrow analysis of the primary recipient mice 4 months after transplantation. Left, the graph represents the percentage of donor CD45.1+ bone marrow chimerism. Middle, Graph represents the number of donor CD45.1+ derived Long-Term HSC (LT-HSC: LSK FLK2^neg^, CD48^neg^, CD150^pos^) for each condition. Right, the graph represents the integrated MFI of total intracellular ROS in HSC for each condition relative to 0Gy-HSC. *n* = 5 mice per condition. (C) Bone marrow analysis of the secondary recipient mice 4 months after transplantation. Left, the graph represents the number of donor CD45.1+ derived LT-HSC for each condition. Right, the graph represents the percentage of 20 mGy irradiated donor CD45.1+ derived HSC with high mtROS in the presence or in the absence of 4-PBA. Dotted line represents the value of the control 0Gy. n = 4–6 mice per condition. (D) Left, Splicing of *Xbp1*mRNA in the indicated donor CD45.1+ HSC sorted from the bone marrow of primary recipient mice. An 18S PCR was performed as a cDNA quality and quantity control. Right, the graph represents the ratio of Sp*Xbp1* to USp*Xbp1*. N = 2–5 mice. (E) Left, Splicing of *Xbp1* mRNA in the indicated donor CD45.1+ HSC sorted from the bone marrow of secondary recipient mice. Right, the graph represents the ratio of *SpXbp1* to *USpXbp1*. n = 1–2 mice. Data are represented with mean ± SEM or min to max box-and-whisker. Statistical significance was assessed using a Kruskal-Wallis was used for statistical analysis (B and C). ∗*p* ≤ 0.05; ∗∗*p* ≤ 0.01.

We then studied the effect of 4-PBA pre-treatment on the long-term hematopoietic repopulation capacity of 20 mGy-HSC in primary and secondary recipient mice. CD45.1 LSK cells were transplanted into CD45.2 congenic recipient mice that had been lethally irradiated (9.5 Gy) for a complete myeloablation (Figure 5A). Four months post-transplantation, CD45.1 donor cells had entirely reconstituted hematopoiesis, as indicated by a chimerism exceeding 95%. (Figure 5B, left). The number of CD45.1-HSC was similar in all groups, and these HSC were quiescent (Figure 5B, middle; Figure S5C). However, CD45.1-HSC in mice transplanted with 20 mGy-LSK showed increased total ROS levels, while mice transplanted with 4-PBA pre-treated 20 mGy-LSK did not show this increase (Figure 5B, right panel). Notably, the ROS increase in the 20 mGy HSC did not induce apoptosis (Figure S5D). After secondary transplantation of BM from mice firstly transplanted with 20 mGy-LSK, we found a similar cellularity in all groups (Figure S5E), associated with a decrease in the number of CD45.1-HSC (Figure 5C left) and a high level of mtROS (Figure 5C right). In contrast, after secondary transplantation of BM from mice firstly transplanted with 20 mGy-LSK pre-treated with 4-PBA, we observed an increased number of CD45.1-HSC (Figure 5C left; Figure S5F) without any increase in mtROS in these CD45.1-HSC (Figure 5C, right). Finally, we investigated whether ER homeostasis was restored. Since IRE1α-XBP1 signaling is active in HSC at steady state,^13^ *Xbp1* mRNA splicing was monitored in CD45.1-HSC purified from the BM of primary and secondary mice (Figure S5G). Most *Xbp1* mRNA was spliced, and the ratio between spliced and unspliced *Xbp1* mRNA was increased in CD45.1-HSC purified from mice transplanted with 20 mGy-LSK compared to CD45.1-HSC isolated from mice transplanted with 0Gy-LSK (Figure 5D). This IRE1α-Xbp1 signaling was still higher in CD45.1-HSC isolated from the BM of secondary transplanted mice, and 4-PBA pretreatment abolished the increase in *Xbp1* splicing in HSC (Figure 5E). Taken together, these results show that 20 mGy irradiation of HSC induces persistent ER stress and ROS accumulation in HSC, leading to impaired long-term maintenance of the HSC pool despite the activation of the IRE1α-Xbp1 pathway, and that 4-PBA-mediated ER stress inhibition rescues the 20 mGy irradiated HSC pool.

### Endoplasmic reticulum stress-induced reactive oxygen species leads to heritable hypomethylation of DNA, partially reversed by the pre-treatment of hematopoietic stem cells with 4-phenylbutyric acid

The functional alterations observed in 20 mGy-HSC may be due to genetic and/or epigenetic modifications. Therefore, we investigated any stable chromosomal aberrations, any irradiation-associated mitochondrial DNA deletion,^38^^,^^39^ and any changes in chromatin accessibility or DNA methylation (Figure S6A). We did not observe chromosomal aberrations in LSK isolated from the primary recipient mice transplanted with 20 mGy-LSK and 0Gy-LSK (Figure S6B upper panel), nor in their peripheral blood lymphocyte B cells (Figure S6B lower panel). In addition, no increased mitochondrial DNA deletion could be detected (Figure S6C). As oxidative stress plays a role in epigenetic modifications,^40^ we assessed whether 20 mGy irradiation could induce long-term changes in chromatin accessibility by ATAC-seq. Differential analysis between 20 mGy-HSC and 0Gy-HSC isolated from the BM of primary recipient mice showed no global change in chromatin accessibility between the two groups (only 6 regions differentially accessible, log2FoldChange1 and adjusted *p*-value ≤0,05, out of 114,744 regions identified) (Figure S6D), indicating that 20 mGy irradiation did not alter long-term genome-wide chromatin accessibility.

**Figure 6:**
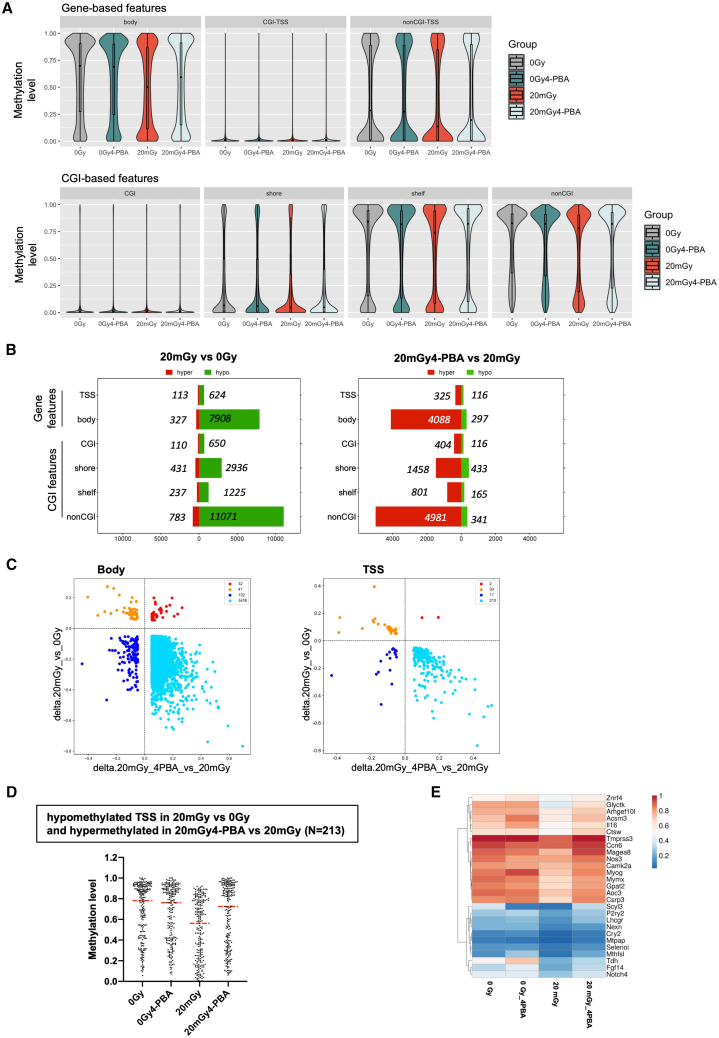
ER stress-induced ROS leads to the heritable hypomethylation of DNA, partially reversed by the pre-treatment of HSC with 4-PBA (A) Violin and boxplot showing DNA methylation levels of different genomic regions in indicated HSPCs isolated from the bone marrow of primary recipient mice 4 months after transplantation. Top, Gene-based feature was defined based on Gencode, and TSS is defined as ±500bp from the transcriptional start site. Bottom, CGI (CpG island) were sourced from the UCSC database, shores were defined as 2 kb on each side of CGI, shelves 2 kb on each side of the shores, and non-CGI 100 kb windows outside CGI features. (B) Bar plots show the number of differentially methylated regions (DMR, q-value ≤0.05, delta >0.05) in gene features and CGI-based features comparing 20 mGy vs. 0Gy-HSPC (left) and 20 mGy+4-PBA vs. 20 mGy-HSPC (right). (C) Correlation scatterplot between statistically significant DNA methylation changes in 20 mGy vs. 0Gy (y axis) and 20 mGy+4-PBA vs. 20 mGy (x axis) in gene body (left) and TSS (right). (D) DNA methylation levels at hypomethylated TSS in 20 mGy vs. 0Gy-HSPC and hypermethylated in 20mGy4-PBA vs. 20 mGy-HSPC. Red line represents the median value. *N* = 213. (E) Heatmap of DNA methylation levels of TSS hypomethylated in 20 mGy vs. 0Gy-HSPC and hypermethylated in 20mGy4-PBA vs. 20 mGy-HSPC of genes associated with mitochondrial metabolism and calcium signaling.

We then investigated genome-wide DNA methylation in HSPC isolated from BM of primary recipient mice transplanted with 20 mGy-LSK and 0Gy-LSK pretreated or not with 4-PBA (0Gy-HSPC, 0Gy4-PBA-HSPC, 20 mGy-HSPC, 20mGy4-PBA-HSPC). The distribution of DNA methylation levels integrated across functional domains followed the well-established patterns observed in mammalian genomes, including low levels of DNA methylation at gene TSS and CpG islands (CGI), bimodal in shores (up to 2 kb from the CGI) and shelves (from 2 kb to 4 kb from the CGI), and high levels in gene bodies and in open sea regions (non-CGI). Significant DNA hypomethylation was observed in 20 mGy-HSPC compared to 0Gy-HSPC, affecting most of the genomic features, i.e., TSS and gene body regions, shelves, and open sea regions. While there was no significant difference between 0Gy and 0Gy4-PBA-HSPC, pretreatment with 4-PBA partially reverted DNA hypomethylation of 20 mGy HSPC (Figure 6A).

A supervised differential methylation analysis revealed widespread hypomethylation in 20 mGy HSPC compared to 0Gy HSPC in both gene-based features (8,532 hypomethylated regions out of a total of 8,972 DMR identified) and CGI-based features (15,882 hypomethylated regions out of a total of 17,443 DMR identified), mainly located in gene bodies and open sea regions, respectively (Figure 6B, right panel). Pre-treatment with 4-PBA significantly increased DNA methylation in both gene-based (4,413 out of 4,826 DMR) and CGI-based features (7,644 out of 8,699 DMR) (Figure 6B, left panel). Considering gene-based features, the majority of these DMR showed a strong negative correlation with DNA methylation changes in the 20 mGy irradiated sample, including 3,416 gene bodies and 213 TSS (Figure 6C). Interestingly, among genes in which DNA hypomethylation at TSS was reversed by 4-PBA pretreatment (Figure 6D), we identified several genes encoding key regulators of mitochondrial metabolism and Ca2+ signaling, such as *P2ry2* and *Camk2a* (Figure 6E). Altogether, these results showed that ER stress generated by 20 mGy irradiation of HSC did not induce major changes in chromatin accessibility but resulted in increased DNA hypomethylation of HSPC, which can be partially reversed by pre-treatment with 4-PBA.

## Discussion

Biological and epidemiological studies on the effects of low-dose radiation on health are poorly understood, although increasing evidence suggests an association between such exposure and elevated risk of leukemia.^41^^,^^42^ At the cellular level, ionizing radiation (IR) can cause nuclear and mitochondrial DNA damages including oxidation and double-strand breaks (DSBs), through direct and indirect action of ROS, leading to genetic mutations that can transform normal HSC.^43^ In our study, exposure of quiescent HSC to a single low-dose of 20 mGy *ex vivo* did not induce nuclear DSB,^3^ chromosomal abnormalities, or mitochondrial DNA deletion. However, it triggered immediate, persistent, and heritable ER stress, which promoted mitochondrial dysfunctions and long-term increase in mitochondrial ROS, ultimately affecting global HSC DNA methylation and a long-term reduction of the HSC pool. These results indicate that ER stress acts as the primary driver of HSC dysfunction after 20 mGy irradiation, with mt ROS acting downstream to mediate long-term functional impairment. Pre-Treatment with 4-PBA successfully abolished initial ER stress, inhibited oxidative stress, preventing deleterious effects in 20 mGy-HSC; partially reversed global DNA hypomethylation and restored HSC maintenance. NAC pre-treatment by scavenging ROS can also prevent long-term HSC defects.^3^ Together, these results indicate a mechanistic link between early ER stress, mitochondrial redox changes, and long-term chromatin remodeling in 20 mGy-HSC and their impaired maintenance. Whether directly targeting mitochondrial oxidative stress or other pathways can also contribute to long-term epigenetic changes remains to be elucidated.

Interestingly, we show that the ER stress response in 20 mGy-HSC is dependent on their physiological state. Low-dose *ex vivo* irradiation of quiescent HSC activates an immediate ER stress, leading to an mtCa^2+^ overload that is relayed by ERO1α and mtROS production. In quiescent 20 mGy-HSC, the initial ER stress response activated the ERAD pathway, which, together with the previously identified pathways, i.e., autophagy and activation of the KEAP-NRF2 pathway, resolves acute oxidative and calcium stress, highlighting the capacity of quiescent 20 mGy-HSC to cope with transient insults. This response occurs without the activation of the canonical UPR, reflecting a selective engagement of ER quality-control mechanisms dependent on stress intensity and duration. Consistently, ERAD can function independently of IRE1α, with SMURF1 promoting ERAD and limiting ER stress via the KEAP1–NRF2 pathway without activating canonical UPR branches.^44^ Moreover, the Sel1L-Hrd1 ERAD complex degrades IRE1α under mild stress, thereby preventing excessive UPR activation.^45^ In contrast, when these cells exit quiescence and start to proliferate, ER stress persists and activates distinct adaptative pathways, including AMPK and selective activation of the eIF2α-ATF4 branch of the UPR, promoting metabolic adaptation and survival. The choice of UPR pathway depends on the degree of ER luminal Ca^2+^ depletion: mild depletion preferentially engages IRE1α-Xbp1, whereas more sustained or severe depletion favors ATF4 activation, followed by ATF6 engagement.^46^ Moreover, studies using strong cytotoxic stress, such as 5-FU treatment, show that pharmacological interventions enhancing ATF4 activity can restore HSC function, supporting that ATF4 mediates an adaptive response under sustained ER stress.^47^ Studies in Drosophila with mitochondrial dysfunction and mtROS that specifically activate the eiF2α-ATF4 branch of UPR, whereas other UPR branches remained inactive^48^ further reinforcing that ATF4 is engaged under conditions of prolonged or intensified ER stress. In proliferative HSC, ATF4 activation is associated with increasing both mitochondrial mass and mitochondrial membrane potential, as well as producing ATP through OXPHOS. Of note, whereas proliferative 20 mGy-HSC do not produce more mitochondrial ATP than 0Gy-HSC, they produce more glycolytic ATP. Given the increased mtROS in proliferative 20 mGy-HSC, their mitochondrial function may be compromised, and these cells may increase glycolysis to rapidly produce ATP when mitochondrial function is impaired. In this context, AMPK activation likely reflects a metabolic adaptation to ER stress and mitochondrial dysfunction, rather than classical energy deprivation. The concurrent activation of classical ER stress markers—including BIP upregulation, ERAD activation, and mitochondrial Ca^2+^ and ROS accumulation—strongly suggests that eIF2α-ATF4 activation in proliferative 20 mGy-HSC is driven by ER stress. This indicates that the metabolic and proliferative adaptations observed are specifically coordinated by ER stress signaling, rather than by other ISR pathways. In proliferative 20 mGy-HSC, this selective activation of ATF4 seems to facilitate a metabolic switch to glycolysis, allowing cell proliferation. Functional assessment of these proliferative HSC revealed a reduction in their long-term maintenance and clonogenic potential, suggesting that although the eIF2α-ATF4 branch is an adaptive response to ER stress that may limit apoptosis, it cannot preserve HSC functional integrity over time. Both AMPK and ATF4 contribute to stress adaptation by promoting survival through the removal of damaged organelles and proteins via autophagy.^49^ The eIF2α-ATF4 pathway can also initiate an autophagy gene transcription program in response to ER stress through the formation of an ATF4-CHOP heterodimer.^50^^,^^51^ In proliferative 0Gy-HSC, autophagy is activated by mechanisms that are independent of the eIF2α-ATF4 pathway to sustain their homeostasis. In contrast, proliferative 20 mGy-HSC did not show active autophagy or increased expression of genes involved in the autophagosome formation, elongation, and function, suggesting that the absence of active autophagy in proliferative 20 mGy-HSC may be due to the dysregulation of mitochondrial respiration.^52^

Four months after the transplantation of 20 mGy-LSK, i.e., when hematopoietic homeostasis is restored, quiescent HSC and their progeny displayed a persistent ER stress, which triggered the activation of the IRE1α-Xbp1 branch of UPR associated with a decreased HSC pool after secondary transplantation. This decrease, together with our previous result^3^ showing that 20 mGy-irradiated HSCs show a 5-fold reduced contribution compared to 0 mGy-HSCs when competing with endogenous HSCs of Rag2^−/−^γc^−/−^cKit^W/v^ mice, suggests an impaired HSC self-renewal. In steady-state conditions, the IRE1α-Xbp1 and the eIF2α-ATF4 branches of UPR are required for HSC function, as the deletion of IRE1α or ATF4 in HSC resulted in impaired repopulation capacity of HSC.^12^ In stress conditions such as acute HSC injury induced by LPS treatment, the IRE1α-Xbp1 branch of the UPR is activated and promotes survival of functional HSC.^13^ However, MARCH5 protein deficiency in HSC permanently disrupts ER function without mitochondrial dysfunction leading to ER stress-mediated mitochondrial apoptosis via IRE1α-Xbp1 activation and to loss of hematopoietic homeostasis.^14^ In addition, in HSC deficient for DPPA5, which maintains low level of ER stress, ER stress induces the activation of the IRE1α -Xbp1 branch of UPR and mitochondrial mediated apoptosis of these HSC.^15^ In conclusion, our study showed differential ER stress responses of 20 mGy-HSC when they are quiescent, when they proliferate and when their progeny is quiescent after transplantation. These ER stress responses protect HSC from apoptosis, but the IRE1α-Xbp1 activation resulting from persistent ER stress finally leads to HSC metabolic dysfunctions, which contribute to their impaired maintenance.

Epigenetics is an important determinant of HSC fate as HSC behavior is determined by epigenetic configuration.^53^ Furthermore, several cellular stress-induced epigenetic changes affect the homeostatic and regenerative functions of HSC and are considered as key effectors driving HSC aging.^54^ Indeed, cumulative changes in DNA methylation, chromatin accessibility, histone modifications, or derepression/mobilization of retroelements have been observed in aged HSC.^55^^,^^56^^,^^57^^,^^58^ These epigenetic changes, together with high mtROS, may lead to the functional decline of aged HSC and an increased propensity for neoplastic transformation.^59^ Here, we identified an epigenetic signature in quiescent HSC derived from transplanted 20 mGy-LSK, induced by ER stress and characterized by global DNA hypomethylation without changes in chromatin accessibility, suggesting that histone modifications are not directly involved. These epigenetic changes are associated with persistently higher levels of mtROS and HSC exhaustion. In HSPC, a loss of global DNA methylation due to loss of DNA methyltransferase 1 (DNMT1) has been observed after^56^ Fe-ion irradiation exposure.^60^ Furthermore, deletion or hypomorphic loss of function of *Dnmt1* in mouse impairs HSC self-renewal capacity.^61^^,^^62^ Together with our data, these results suggest that a critical threshold of DNA methylation is required to maintain homeostasis of HSC.

S-adenosylmethionine (SAM), the donor of the methyl group for DNMT1, is closely linked to mitochondrial function.^63^ Its decreased availability due to mitochondrial dysfunction, which can lead to DNA hypomethylation, impair protein synthesis, and exacerbate redox state, exacerbating ER stress and oxidative stress. Interestingly, supplementation of methyl donors attenuates the effects of radiation and is associated with normal patterns of DNA methylation.^64^ Given the intricate relationship between SAM metabolism, DNMT activity, and UPR, further studies are needed to explore the cross-talks between these three pathways after low-dose irradiation of HSC.

Targeting the ER stress with 4-PBA prior to irradiation can partially reverse the global DNA hypomethylation observed in transplanted 20 mGy-HSC and can prevent HSC dysfunctions. Of note, 4-PBA can reverse DNA hypomethylation at the promoters of several genes encoding key regulators of metabolism and Ca2^+^ signaling, such as *Tdh*, *Glyctk, P2ry2,* or *Camk2a,*^65^ which might explain its action. Indeed, the dysregulation of Ca^2+^ signaling by epigenetic mechanisms has been proposed as a key event in several diseases, including carcer.^66^^,^^67^ However, 20 mGy IR leads to the reduced DNA methylation of HSC across the entire genome, and the partial reversal of DNA hypomethylation by 4-PBA affects gene bodies, CGI shores, and non-CGI regions, all of which contain retrotransposons. Retrotransposons DNA hypomethylation may lead to their activation, which was observed in HSC after TBI,^68^ thus our results strongly suggested an activation of retrotransposons or repetitive sequences rather than changes in specific promoters. Furthermore, retrotransposons transcription, which plays a critical role in HSC activation after stress^69^ and that enhances haematopoietic regeneration after chemotherapy.^70^ Thus, studying retrotransposon transcription in 20 mGy-HSC might indicate the relationship between hypomethylation and the biological properties of 20 mGy-HSC.

In conclusion, our data suggest mechanisms by which ER stress triggered by a single dose of 20 mGy induces persistent mitochondrial dysfunction and global DNA hypomethylation, which ultimately drives long-term impaired maintenance of 20 mGy-HSC. These findings underscore the importance of ER stress in the long-term detrimental effects of low-dose radiation on HSC and suggest that targeting ER stress may offer a promising avenue for preserving HSC function after low-dose radiation exposure.

### Limitations of the study

There are two limitations of our study.

First, we only use pharmacologic inhibitors and do not use genetic evidence, such as ATF4 knockout mice, to study the molecular basis of the 20 mGy irradiation of HSC.

Second, we show that low-dose irradiation induces long-term global DNA hypomethylation associated with oxidative and ER stress, leading to impaired long-term maintenance of HSC. Treatment with 4-PBA, acting through its assistance in protein remodeling, restores the long-term status of 20 mGy irradiated HSC, but results only in a partial reversal of DNA hypomethylation. This suggests that additional mechanisms, beyond epigenetic regulation, may contribute to the long-term defects in HSC function. However, these alternative pathways were not investigated in the present study.

## Resource availability

### Lead contact

For any further information and requests for resources and reagents, please contact Nathalie Gault nathalie.gault@cea.fr, who will handle them.

### Materials availability

No new unique reagent was generated in this study.

### Data and code availability

• Data reported in this article will be shared by the lead contact upon request.
• ATAC-seq, methylome, and transcriptome data have been deposited in Gene Expression Omnibus (GEO) and are publicly available as of the date of publication. Accession numbers are listed in the key resources table. All data reported in this article will be shared by the lead contact upon request.
• This article does not report original code.
• Any additional information required to reanalyse the data reported in this article is available from the lead contact upon request.

## Acknowledgments

We thank Dr. S. Vincent-Naulleau and the staff of Uniivo for their care of the animals housed in the institute’s animal facility. We also appreciated V. Menard for her assistance with irradiations and dosimetry. The authors thank the GenomEast platform (IGBMC Strasbourg, France) for their excellent support with ATACseq experiments, and to S. Le Gras for her help in analyzing the ATACseq results. We are grateful to IntegraGen (Evry-Courcouronnes, France) for their support with the methylome experiments. We thank Life & Soft (Fontenay-aux-Roses) for their advice on transcriptomic analysis. Finally, we thank Dr. Françoise Pflumio, Dr Marie-Catherine Vozenin, and Dr Claire Francastel for their helpful discussions on the article. This work was supported by funding from IRBIO/10.13039/501100006489CEA program, 10.13039/501100001677INSERM, and 10.13039/501100016036Electricité de France (EDF).

## Author contributions

S.G.M. and F.F. performed experiments, took part in discussions about the results, and wrote the article; D.L. performed transcriptomic experiments and participated in the reading of the article; V.B., S.D., and N.D. provided experimental assistance; P-H.R. wrote the article and took part in discussions on the work; and N.G designed, supervised, and performed all experiments, prepared Figures, and wrote the article.

## Declaration of interests

The authors declare no competing financial interests.

## STAR★Methods

### Key resources table

**Table:** 

REAGENT or RESOURCE	SOURCE	IDENTIFIER
**Antibodies**		

Hematopoietic lineage labeling Cocktail, anti-mouse, Biotin	Miltenyi Biotec	Cat# 130-092-613; RRID:AB_1103214
Direct Lineage Cell Depletion kit	Miltenyi Biotec	Cat# 130-110-470
APC anti-mouse CD117 Antibody	BioLegend	Cat# 105812 (clone 2B8); RRID:AB_313221
APC/Cyanine 7 anti-mouse CD117 Antibody	BioLegend	Cat# 105826 (clone 2B8); RRID:AB_16262278
CD117 (c-kit) Monoclonal Antibody, Alexa Fluor™ 700	eBioscience™	56-1172-82 (clone ACK2); RRID:AB_657583
PE/Cyanine7 anti-mouse Ly-6A/E (Sca-1) antibody	BioLegend	Cat# 108114 (clone D7); RRID:AB_493596
Ly-6A/E (Sca-1) Monoclonal Antibody, perCP-Cyanine5.5	eBioscience™	Cat# 45-5981-82 (clone D7); RRID:AB_914372
Brillant Violet 785™ anti-mouse Ly-6A/E (Sca-1) Antibody	BioLegend	Cat# 108139 (clone D7); RRID:AB_256557
PE anti-mouse Ly-6A/E (Sca-1) Antibody	BioLegend	Cat# 122508 (clone E13-161.7); RRID:AB_756193
Biotin Rat Anti-mouse Ly-6A/E	BD Biosciences	Cat# 553334 (clone E13-161.7); RRID:AB_394790
CD34 Monoclonal Antiboy, efluor™ 450	eBioscience™	Cat# 48-0341-82 (clone RAM34); RRID:AB2043837
FITC Rat anti-mouse CD34	BD Biosciences	Cat# 553733 (clone RAM34); RRID:AB_395017
PE/Cyanine7 anti-mouse CD48 Antibody	BioLegend	Cat# 103424 (clone HM48-1); RRID:AB_2075049
APC anti-mouse CD48 Antibody	BioLegend	Cat# 103412 (clone HM48-1); RRID:AB_571997
APC anti-mouse CD135 Antibody	Biolegend	Cat# 135310 (clone A2F10); RRID:AB_2107050
PE Rat Anti-Mouse CD135	BD Biosciences	Cat# 553842 (clone A2F10); RRID:AB_395079
Brillant Violet 421™ anti-mouse CD135 Antibody	BioLegend	Cat#135314 (clone A2F10); RRID:AB_2562339
PE Mouse Anti-Mouse CD45.1	BD Bioscience	Cat# 553776 (clone A20); RRID:AB_395044
Pacific Blue™ anti-mouse CD45.1 Antibody	BioLegend	Cat# 110722 (clone A20); RRID:AB_492866
APC anti-mouse CD150 (SLAM) Antibody	BioLegend	Cat# 115910 (clone TC15-12F12.2); RRID_AB_493460
CD150 Monoclonal Antibody, FITC	eBioscience	Cat# 11-1501-82 (clone 9D1); RRID:AB_465209
BD Horizon™ BUV395 Streptavidin	BD Bioscience	Cat# 564176; RRID:AB_2869553
BiP Rabbit mAB (PE Conjugate)	Cell Signaling Technology	Cat# 14464 (clone C50B12); RRID:AB_2798486
Rabbit mAB IgG XP^R^ Isotope Control (PE Conjugate)	Cell Signaling Technology	Cat# 5742 (clone DA1E); RRID:AB_10694219
Phospho-AMPK alpha-1,2 (Thr183, Thr172) Polyclonal Antibody	ThermoFischer	Cat# 44-1150G; RRID:AB_2533585
Dylight 649 Donkey anti-rabbit IgG minimal X-reactivity Antibody	BioLegend	Cat# 406406; RRID:AB_1575135
Anti-Nrf2 Antibody	Abcam	Cat# ab62352 (clone EP1808Y); RRID:AB_944418
ATF4 Recombinant Rabbit Monoclonal Antibody	Thermo Fisher Scientific	Cat# MA5-32364 (clone SD20-92); RRID:AB_2809645
LC3B Antibody	Cell Signaling Technology	Cat# 2775; RRID:AB_915950
Goat anti-Rabbit IgG (H+L), Secondary Antibody, Alexa Fluor™ 594	Thermo Fisher Scientific	Cat# A-11012; RRID:AB_141359
eIF2α XP Rabbit mAb	Cell Signaling Technology	Cat# 5324 (clone D7D3); RRID:AB_10692650
Phospho-eIF2α (Ser151) Rabbit mAb	Cell Signaling Technology	Cat# 3597 (clone 119A11); RRID:AB_390740
Vinculin XP rabbit mAb	Cell Signaling Technology	Cat# 13901 (clone E1E9V); RRID:AB_2728768
GAPDH Rabbit mAb	Cell Signaling Technology	Cat# 2118 (clone 14C10); RRID:AB_561053
Ki-67 Monoclonal Antibody, FITC	eBioscience™	Cat# 11-5698-82 (clone SolA15); RRID:AB_11151330
CD150 Monoclonal Antibody, super Bright™	Thermo Fisher scientific	Cat# 62-1502-82 (clone mShad150); RRID:AB_2734882

**Chemicals, peptides, and recombinant proteins**		

Mouse Recombinant Flt3/Flk-2 Ligand	STEMCELL Technologies	Cat# 78011
Mouse Recombinant SCF	STEMCELL Technologies	Cat#78064
Mouse Recombinant IL-11	STEMCELL Technologies	Cat# 788026.3
4-Phenylbutyric acid 99%	Sigma-Aldrich	Cat# P21005
Bafilomycine A1	Sigma-Aldrich	Cat# 19148
StemSpan™ SFEM Culture media	STEMCELL Technologies	Cat# 09650
Mouse Recombinant IL-4	STEMCELL Technologies	Cat# 78047
Cell-Tak™	BD Biosciences	Cat# 354240

**Critical commercial assays**		

Annexin V apoptosis detection	eBioscience™	Cat# 12770170
CM-H2DCFDA	Invitrogen	Cat# C6827
MitoSOX™ Red	Invitrogen	Cat# M36008
CellEvent™ Caspase-3/7 Detection Reagebts	Invitrogen	Cat#C10423
BD Cytofix/Cytoperm™ Fixation/Permeabilization Kit	BD Biosciences	Cat# 554714
Fluo-3AM, Calcium Indicater	Invitrogen	Cat# F1242
Rhod-2AM, cell permeant	Invitrogen	Cat#R1244
Pluronic F-127	Sigma-Aldrich	Cat# P2443
MitoTracker™ Dyes for mitochondria labeling	Invitrogen	Cat# M7514
Tetramethylrhodamine, Ethyl Ester, Perchlorate (TMRE)	Invitrogen	Cat# T669
Seahorse XF Real-Time ATP Rate Assay Kit	Agilent	Cat# 103591-100
MitoBiogenesis™ Flow Cytometry Kit	Abcam	Cat# ab168540
PROTEOSTAT Aggresome detection kit	Enzo Life Science	Cat# ENZ-51035
OxylICC™ Oxidized Protein Detection kit	Merck Millipore	Cat# S7350
12-230 kDA separation Module	Bio-Techne	Cat# SM-W001
Anti-Rabbit Detection Module	Bio-Techne	Cat# DM-001
Fluorescent 5X Master Mix 1	Bio-Techne	Cat# PS-FL01-8
COmplete™ Protease inhibitor Cocktail, Tablets	Merck Millipore	Cat# 11836145001
Phosphatase Inhibitor Cocktail1 DMSO solution	Sigma-Aldrich	Cat# P2850
Phosphatase Inhibitor Cocktail	Sigma-Aldrich	Cat# P5726
RNeasy Plus Micro Kit	QIAGEN	Cat# 74034
Transcriptase inverse SuperScript™ IV	Invitrogen	Cat# 18090-050
Master Mix PCR Power SYBR™ Green	Thermo Fischer Scientific	Cat# 4367659
Clariom™ S Assay, mouse	Thermo Fischer Scientific	Cat#902930
MethoCult™ M3434 methylcellulose -Based medium (Mouse)	STEMCELL Technologies	Cat# Methocult™ GF M3434
EasySep™ Mouse B cell Isolâtion kit	STEMCELL Technologies	Cat# 19854
Direct Lineage cell depletion Kit, mouse	Miltenyi Biotec	Cat# 130-110-470
21XMouse Multicolor FISH Probe for Mouse chromosomes	Metasystems Probes	Cat# D-0425-120)DI
DAPI/Antifade	Metasystems Probes	Cat# D-0902-500-DA
QIAamp DNA Micro Kit	QIAGEN	Cat# 56304
Phusion High-Fidelity DNA Polymerase	New England Biolabs	Cat# M0530
ReadyMix PCR REDExtract-N-Amp^TMTM^ Ready-to-use 2X	Sigma-Aldrich	Cat# R4775

**Deposited data**		

ATAC-seq	this paper	GEO: GSE281654
Methylome	this paper	GEO: GSE281655
Transcriptome	this paper	GEO: GSE286157

**Experimental models: Organisms/strains**		

C57BL/6JRj wild-type mice	JANVIER Labs	https://janvier-labs.com/fiche_produit/2-c57bl-6jrj/#onglet__1
C57BL/6JRj wild-type mice	our animal facility	N/A
Ly5.1 mice	Charles River	#494 https://www.criver.com/products-services/find-model/ly51-mouse?region=29
Ly5.1xC57BL/6JRj mice	our animal facility	N/A

**Oligonucleotides**		

XBP1 3S forward: 5’-AAACAGAGTAGCAGCGCAGACTGC-3'	Eurofins	N/A
XBP1 12S reverse: 5’-TCCTTCTGGGTAGACCTCTGGGAG-3'	Eurofins	N/A
XBP1 intF forward 5’-GATCCTGACGAGGTTCCAGA-3'	Eurofins	N/A
XBP1 intR reverse: 5’-ACAGGGTCCAACTTGTCCAG-3'	Eurofins	N/A
18S F1 forward: 5’-GTAACCCGTTGAACCCCATT-3’	Eurofins	N/A
18S R1 reverse: 5’-CCATCCAATCGGTAGTAGCG-3’	Eurofins	N/A
qRT-PCR oligos: see Table S1	this paper	N/A

**Software and algorithms**		

ProteinSimple's Compass software v5.0.1	Bio-Techne	https://www.bio-techne.com/resources/instrument-software-download-center/compass-software-simple-western
Encode ATAC-seq pipeline v1.5.1.	Encode	https://github.com/ENCODE-DCC/atac-seq-pipeline
BS-Seeker2	Guo et al.^71^	https://guoweilong.github.io/BS_Seeker2/index.html
methylKit package	Bioconductor	https://bioconductor.org/news/bioc_3_22_release/
Transcriptome Analysis Console (TAC) Software	Thermo Fisher	https://www.thermofisher.com/fr/fr/home/life-science/microarray-analysis/microarray-analysis-instruments-software-services/microarray-analysis-software/affymetrix-transcriptome-analysis-console-software.html
GSEA software v4.1.0	Broad Institute	https://www.gsea-msigdb.org/gsea/index.jsp
GraphPad Prism v10	GraphPad Software	Version 10.3.1
FlowJo software v10	BD	Version 10.8.2
ImageJ software	ImageJ	https://imagej.net/software/imagej/
Seahorse Analytics software	Agilent	https://www.agilent.com/en/product/cell-analysis/real-time-cell-metabolic-analysis/xf-software/agilent-seahorse-analytics-787485
ISIS® V4 software	Metasystems	https://metasystems-international.com/

### Experimental model and study participant details

#### Mice

C57BL/6 mice, aged 8–12 weeks, were bred and maintained in a specific-pathogen-free facility (EU Agreement number E92-032-02) or purchased from Janvier laboratory (male CD45.2^+^ C57BL/6JRj mice) or Charles River laboratory (female CD45.1^+^ C57BL/6-Ly5.1 mice). CD45.1^+^CD45.2^+^ C57BL/6 (F1) mice were generated by crossing these two strains in our animal facility. All experimental procedures adhered to the European Community Council Directive (EC/2010/63) and were approved by the institutional ethics committee (APAFIS#23783-2020012414542835 v1). Animal handling complied with institutional guidelines and French Ministry of Agriculture regulations.

#### Irradiation

Irradiations were performed on two different GSR-D1 irradiators (Gamma-Service Medical GmbH company). For low dose irradiation, the first GSR-D1 is a self-shielded irradiator with three sources of ^137^Cesium with a total activity around 30.03 TBq (April 2019) and which emits gamma rays. Cells were irradiated in Stemspan medium at 20mGy with an output 9 mGy/min taking the radioactive decrease into account. Prior irradiation, dosimetry was performed.

A cylindrical ionizing chamber 31010 by PTW was used as the recommendation of the AAPM’S TG-61. This ionizing chamber has a cavity of 0.125 cm^3^ calibrated in ^137^ Cesium air kerma free in air at the PTB reference facility number 1703558. The polarity and the ion recombination were measured for this ^137^Cesium source. Each measurement was corrected by the KTP factor to take the variation of temperature and atmospheric pressure into account.

For radio-induced myeloablation needed for transplantation conditioning of recipient mice, GSR-D1 is a self-shielded irradiator with four sources of ^137^Cesium with a total activity around 180.28 TBq (March 2014) and which emits gamma rays (662 KeV). Mice were irradiated at a single dose of 9.5 Gy with an output of 1.03 Gy/min taking the radioactive decrease into account. Mice were in specific box during the irradiation. Prior irradiation, dosimetry was performed with this box. For dosimetry, this ionizing chamber has a cavity of 0.125 cm^3^ calibrated in ^137^ Cesium dose water with the PTB reference facility number 1703557.

#### LSK cell sorting from bone marrow for transplantations and 4-PBA treatment

Bone marrow cells were harvested from humerus, femurs, tibias and pelvis using a syringe filled with DPBS (Gibco). Following red blood cells lysis with an ammonium chloride solution (STEMCELL™) for 10 min, bone marrow cells were filtered through a 70μm-cell strainer and depleted of mature hematopoietic cells using direct Lineage Cell Depletion kit (Miltenyi Biotec). LSK (Lineage^neg^, Sca-1^+^, c-kit^+^) cells were isolated using a BD Influx™ Cell Sorter (BD Biosciences) after staining with PE-conjugated anti-Sca-1 and APC-conjugated anti-c-kit antibodies.

For reconstitution assay (mid- and long-term), CD45.2 recipient received 9.5 Gy lethal irradiation before transplantation with 2.10^4^ sorted CD45.1/CD45.2 LSK that were previously irradiated or not at 20 mGy and pre-treated or not for 1 hour with 1μM 4-Phenyl Butyric Acid (4-PBA).

Four to five months post-transplantation, mice were euthanized for bone marrow analyses including phenotype analysis, HSC sorting for ATAC-seq or methylome analyses and for secondary transplantation. Secondary transplants involved 2 × 10^6^ bone marrow cells from primary recipients with >90% CD45.1/CD45.2 chimerism. Four to five months later, mice were euthanized and bone marrow cells were prepared for phenotypic analysis.

Transplantation was performed via retro-orbital injection under isoflurane anesthesia. TRISULMIX® was administered in drinking water for one-month post-transplantation to prevent infections. Body condition scoring was monitored and scored for 15 days post-irradiation.

### Method details

#### Hematopoietic stem cell (HSC) isolation, culture protocol and inhibitor treatments

HSC (Lineage-^neg^, c-kit^+^, Sca-1^+^, CD135-^neg^, CD48-^neg^) were isolated from lineage-depleted bone marrow cells using a BD ARIA II™ Cell Sorter after staining with PECy7-conjugated anti-Sca-1, APCCy7-conjugated anti-c-kit, PE-conjugated anti-CD135, and APC-conjugated anti-CD48 antibodies.

HSC were either irradiated (20 mGy) or left untreated in StemSpan™ SFEM medium. Following irradiation, cells were seeded at low density (500 cells/cm^2^) into 10–12 mL of complete StemSpan™ SFEM medium supplemented with 100 ng/ml mouse recombinant Flt3L, 50 ng/ml mouse recombinant SCF, and 100 ng/ml mouse recombinant IL-11 and cultured for 2, 4, 5, or 6 days. Post-culture, cells underwent flow cytometry analysis or further HSC sorting for downstream applications.

Hematopoietic stem cells (HSC) were treated with two endoplasmic reticulum (ER) stress inhibitors - 4-phenylbutyric acid (4-PBA) and tauroursodeoxycholic acid (TUDCA) - and an ERO1α inhibitor (EN460) following these protocols.

**4-PBA treatment:** HSC were treated with 1 μM 4-PBA for 1 hour either (i) immediately after isolation and prior irradiation followed by 6 days of culture, or (ii) after irradiation and 6 days with culture, with 4-PBA added one hour before measurement of mitochondrial reactive oxygen species (mtROS) or mitochondrial calcium. The treatment duration was based on our previous experiments with NAC.^3^ The concentration of 4-PBA was optimized by treating lineage-negative bone marrow cells isolated via magnetic-activated cell sorting (MACs) with increasing concentrations (0.1–1 μM). HSC were identified using specific surface markers, in optimization experiments, treated for 1 hour prior to irradiation (20mGy) or sham-treatment, with mtROS measured 30 minutes post-irradiation. Based on these results, experiments, 1 μM 4-PBA was selected for all subsequent experiments.

**TUDCA treatment:** HSC were treated with 60 μM TUDCA^15^ for 1 hour on day 6 of culture prior analysis of mitochondrial reactive oxygen species.

**EN460 treatment:** HSC were treated with 10-25 μM EN460^36^ for 1 hour either (i) on day 6 of culture or (ii) on freshly isolated HSC before exposure to 20mGy prior analysis of mitochondrial calcium.

#### Flow cytometry analyses

Flow cytometry analyses were conducted using a BD FACSLSRII™ flow cytometer (BD Biosciences) on cells isolated directly from bone marrow or after culture. Cells were stained with specific markers for 15 minutes at 4°C to identify different hematopoietic stem and progenitor cell populations using the following immunophenotypic definitions: Long-term HSC (LT-HSC): Lineage^neg^, c-kit^+^, Sca-1^+^, CD135^neg^, CD48^neg^, CD150^+^; Hematopoietic stem cells (HSC): Lineage^neg^, c-kit^+^, Sca-1^+^, CD135^neg^, CD48^neg^ or Lineage^neg^, c-kit^+^, Sca-1^+^, CD135^neg^, CD34^neg^; Hematopoietic stem and progenitor cells (HSPC): Lineage^neg^, c-kit^+^, Sca-1^+^, CD135^neg^.

For bone marrow chimerism analysis in transplanted mice, CD45.2 and CD45.1 antibodies distinguished donor and recipient cells.

##### Cell cycle analysis

HSC were fixed and permeabilized using the BD Cytofix/Cytoperm kit. Cells were resuspended in 100μl of 1X BD Perm/Wash buffer containing 0.5% of Bovine Serum Albumine (BSA, Sigma-Aldrich) and incubated with 20μl of FITC-conjugated Ki-67 for 30 minutes at 4°C. Subsequently, cells were stained with a 2.5 μg/ml Hoechst 33258 solution (Sigma-Aldrich) for DNA content analysis.

##### AMPK activation measurement

HSC were fixed, permeabilized and resuspended as described above. They were incubated with anti Phospho-AMPK alpha-1,2 (Thr172) polyclonal antibody (1:50 dilution) for 2 hours at room temperature. After washing, cells were labeled with a DyLight™ 649 donkey anti-rabbit IgG (1:100 dilution) for 30 minutes at room temperature and then analyzed by flow cytometry. A negative control omitting the primary antibody was included to assess specificity.

To validate flow cytometry findings, HSPC were sorted at day 6 of culture and immediately lysed in 2X RIPA buffer, supplemented with proteases and phosphatase inhibitors. Lysates were prepared for WES analysis by mixing 4 volumes of cell lysates with one volume of 5X fluorescent master mix. Samples were denatured at 95°C for 5 min and 4μl of each sample was loaded. Detection of AMPK (66 kDa), phospho-AMPK (Thr172, 66kDa) and Vinculin (116 kDA) were performed using primary antibodies diluted in the supplied antibody diluent (AMPK and Phospho-AMPK at 1:50; and Vinculin 1:100). Samples, antibodies and reagents were loaded into the WES plate according to the manufacturer’s instructions and the assay was run on the Protein Simple WES system under default settings for the relevant molecular weight range. Signal detection and quantification were performed using Compass for Simple Western software. Results are presented as the ratio of phospho-APMK to total AMPK after normalization to vinculin.

##### Reactive oxygen species (ROS) measurement

For intracellular and mitochondrial ROS detection, HSC were suspended in DPBS1X (Gibco) containing calcium and magnesium, supplemented with 0.5% BSA (Sigma-Aldrich) and incubated respectively with the Molecular Probe™ CM-H2DCFDA™ (10 μM final, 30 minutes at 37°C) or the Molecular Probe™ MitoSOX™ (2 μM final, 30 minutes at 37°C). Cells were washed twice with DPBS w/o calcium and magnesium (Gibco). To define Ros^high^ and ROS^low^ HSC populations, we first determined the median fluorescence intensity of CM-H2DCFDA or MitoSox within the non-irradiated HSC population. HSC with fluorescence above the median were classified as ROS^high^ while those below the median were classified as ROS^low^ (approximately 50% of the population each). This gating window, established in the control condition, was then applied identically to irradiated samples allowing a direct comparison between conditions.

##### Apoptosis

HSC at day 6 of culture were incubated with the CellEvent™ Caspase-3/7 Green Detection Reagent (1/1,000 final dilution) for 40 min at room temperature. Samples were placed on ice until FACS analysis. Donor CD45.1-HSC were incubated with Annexin V binding buffer and stained with Annexin V antibody (eBioscience™ # 12770170) and Ho33342.

##### Cytosolic and mitochondrial *calcium*

HSC were suspended in HBSS1X (Gibco) supplemented with 1% Fetal Calf Serum (Sigma-Aldrich) and incubated 20 minutes at 37°C with either the FLUO-3, AM probe (0.2 μM final concentration) or the RHOD-2, AM probe (177 nM final concentration). The probes were previously reconstituted in 100μl of anhydrous DMSO and 100μl of 20% Pluronic® F-127. Cells were then washed with HBSS (1X) supplemented with 0.1% Fetal Calf Serum and 50 μM verapamil followed by a 20 minutes incubation at 37°C before FACS analysis. To define Ca^2+high^ and Ca^2+low^ HSC populations, we first determined the median fluorescence intensity of FLUO-3AM or RHOD-2AM within the non-irradiated HSC population. HSC with fluorescence above the median were classified as Ca^2+high^ while those below the median were classified as Ca^2+low^ (approximately 50% of the population each). This gating window, established in the control condition, was then applied identically to irradiated samples allowing a direct comparison between conditions.

##### Mitochondrial mass and membrane potential

HSC were suspended in 0.5% BSA/PBS with Ca^2+^ and Mg^2+^ and incubated either with 20 nM MitoTracker™ Green (MTG) or with 25 nM Tetramethylrhodamine, Ethyl Ester, Perchlorate (TMRE) for 30 min at 37°C in the dark. Cells were then washed with PBS before FACS analysis.

To define TMRE^high^ and TMRE^low^ HSC populations, we first established the median fluorescence intensity of TMRE within the non-irradiated HSC population. HSC with fluorescence above the median were classified as TMRE^high^ while those below the median were classified as TMRE^low^ (approximately 50% of the population each). This gating window, established in the control condition, was then applied identically to irradiated samples allowing a direct comparison between conditions.

##### Mitochondrial biogenesis

HSC were fixed in a 4% paraformaldehyde solution for 15 min at room temperature in the dark and analyzed using the MitoBiogenesis™ Flow Cytometry Kit according to the manufacturer’s protocol. Cells were washed and permeabilized in 10% methanol cold medium for 30 min at -20°C. Cells were twice washed, incubated in 50 μl of 1X Blocking Buffer for 15 min at room temperature and labelled with 50μl of primary antibody cocktail solution preparing by mixing 1 μl MTCO1-Alexa® 488 and 1 μl SDHA-Alexa® 647 antibodies in 48 μl 1X Blocking Buffer for 1h prior to FACS analysis.

##### Protein aggregate detection

Aggregated proteins in HSC were detected using the PROTEOSTAT® Aggresome detection kit. Cells were fixed with a 4% formaldehyde solution for 30 min, permeabilized for 30 min on ice in a 1X Assay buffer supplemented with 0.5% Triton X-100 and 3 mM EDTA pH 8 and incubated with the Proteostat® Aggresome detection reagent (1:10,000 dilution) for 30 min at room temperature, protected from light prior to FACS analysis. The proteasome inhibitor MG132 was used as a positive control.

#### ATP assays

ATP production in HSPC suspension cells (harvested at day 6 of culture with a purity greater than 80% as determined by flow cytometry under both 0 Gy and 20mGy conditions; see Figure S2F) was assessed using the Seahorse XF ATP Rate Assay (Agilent*).* The XF sensor cartridge was hydrated overnight in XF calibrant solution at 37 °C. On the day of the assay, XF96 plates were coated with Cell-Tak™, seeded with cells, and centrifuged at 200 × g for 1 min to ensure adherence. The culture medium was then replaced with XF assay medium (pH 7.4) supplement with 25 mM glucose, 1 mM pyruvate and 2 mM glutamine and equilibrated for 45 min at 37 °C in a CO_2_-free incubator. Oligomycin (1.5 μM, Port A) and rotenone/antimycin A (0.5 μM, Port B) were freshly prepared in assay medium and loaded into the sensor cartridge. Basal measurements were recorded prior to sequential inhibitor injections according to the manufacturer’s instructions. ATP production rates from oxidative phosphorylation and glycolysis were calculated using Seahorse Wave software and normalized to cell number per well. Experiments were performed in three independent replicates.

#### Quantification of oxidized proteins

On day 6 of culture, sorted HSC were resuspended in cold methanol for 5 min for fixation. Following washing with DPBS and centrifugation, HSC were plated onto Poly-L-Lysine-coated μ-slides VI (ibidi, #80604). Oxidized proteins in HSC were measured using the OxylCC™ Oxidized Protein detection kit according to the manufacturer’s instructions. Nuclei were stained with 1μg/ml DAPI. Images were using a Leica confocal microscope SPE. The Mean Cy3-fluorescent DNP intensity in HSC cytoplasm was measured after processing the images with the ImageJ software. A minimum of 50 cells per condition was analyzed.

#### ATF4, NRF2 and LC3B immunostaining

Sorted HSC were fixed with 4% paraformaldehyde for 10 min at RT, washed with DPBS and plated onto poly-D-lysine-coated slides. After permeabilization with 0.2% Triton X-100 in DPBS (Ca^2+^/Mg^2+^) for 15 min, non-specific binding was blocked with 5% normal goat serum (NGS) with 0.2% Triton X-100 in DPBS for 1 hour. Cells were then incubated with primary antibodies against ATF4 or NRF2 (1/100 dilution). For LC3B immunofluorescent staining, sorted HSC were either pretreated with 5 nM Bafilomycin A1 or left untreated. Cells were fixed with 100% cold methanol for 5 min at 4°C, washed with DPBS and deposited onto poly-D-lysine-coated slides. After permeabilization with 0.5% saponin in DPBS (Ca^2+^/Mg^2+^) for 15 min, blocking was done with 10% NGS, 0.1% saponin in DPBS (Ca^2+^/Mg^2+^) for 1h. Finally, cells were incubated with LC3B primary antibody (1/250 dilution). For all conditions, the secondary antibody used was Alexa Fluor 594® anti-rabbit and nuclei were stained with DAPI (1μg/ml). Slides were then mounted with Fluoromount-G. Image acquisition was performed with a Leica confocal microscope SPE. The images were processed with ImageJ software. A minimum of 30 cells per condition was analyzed.

#### eIF2α phosphorylation

To determine whether ER stress established at day 6 of culture is associated with eIF2α phosphorylation in 20mGy-HSPC, protein lysates were prepared from sorted 20mGy or 0Gy HSPC using a RIPA buffer supplemented with a cocktail of 1X protease inhibitors and 1X phosphatase inhibitors. Samples were denatured for 5 min at 95°C in the presence of 5X Fluorescent Master Mix, and then automatically loaded into the capillaries, for electrophoretic separation by size using the WES system (protein Simple). Primary antibodies were used at the following dilutions: eIF2α (1:200, 66 kDa), P-eIF2α (1:25, 66 kDA). Protein levels were quantified using the Compass software v5.0.1 which detects chemiluminescent signals and calculates both molecular weight and signal intensity (area). Results are as the ratio of P-eIf2α /eIf2α.

#### Quantitative RT-PCR

Total RNA was extracted from sorted HSC using the RNeasy Plus Micro kit and reverse transcribed with random primers and Superscript IV reverse transcriptase. Quantitative PCR reactions were performed using the Power SYBR™ green Master mix in a StepOne™ Real-Time PCR System (Applied Biosystems). Primer sequences are listed in Table S1.

#### Transcriptome analysis and GSEA

On day 6 of culture, 20mGy- and 0Gy-HSC were immediately lysed in RLT Plus Buffer after cell sorting. Total RNA was extracted according to the manufacturer’s protocol. RNA integrity (RIN score ≥ 7.0) and RNA concentration were assessed with the Agilent 2100 Bioanalyzer (Agilent). For transcriptome analysis, total RNA samples were processed using the GeneChip™ Whole transcript Pico Reagent Kit (Applied Biosystems) and analyzed with the Clariom™ S Assay, mouse. Data were collected and analyzed with the Transcriptome Analysis Console (TAC) Software (ThermoFisher). Gene Set Enrichment Analysis (GSEA) was performed on transcriptomic data obtained using GSEA software (v4.1.0, Broad Institute) with the Hallmark gene set collection from the Molecular Signatures Database (MSigDB). Default parameters were applied, including 1000 permutations and a false discovery rate (FDR) threshold of 0.25. The enrichment score (ES) was calculated for each gene set, and the normalized enrichment score (NES) was used to account for gene set size differences. Significantly enriched pathways were identified based on nominal p-value < 0.05 and FDR q-value < 0.25.

#### Xbp1 splicing

Five ng of total RNA extracted from HSC were reverse transcribed. HSC were isolated by cell sorting either after 6 days of *in vitro* culture, or from the bone marrow of mice 4 months after transplantation (primary and secondary recipients). A first PCR was performed to amplify the cDNA, including the potentially spliced region of Xbp1, using the Xbp1 3S forward primer (5’-AAACAGAGTAGCAGCGCAGACTGC) and the Xbp1 12S reverse primer (5’-TCCTTCTGGGTAGACCTCTGGGAG). Next, 50% of the PCR product was used for a second PCR to distinguish between the spliced and unspliced regions, employing the XBP1 intF forward primer (5’-GATCCTGACGAGGTTCCAGA) and the XBP1 intR reverse primer 5’-ACAGGGTCCAACTTGTCCAG). The PCR products were loaded on a 3% UltraPure™ agarose gel in TBE (Invitrogen) to visualized the 193 bp spliced band and the 219 bp unspliced band. As an internal amplification control, we used the 18S rRNA forward (5’-GTAACCCGTTGAACCCCATT) and reverse (5’-CCATCCAATCGGTAGTAGCG) primers on 1 ng of cDNA. The PCR product was loaded on a 2% agarose gel in TBE (Invitrogen) to visualized the 151 bp band. Gels were scanned using the Amersham™ Typhoon and band intensity was quantified with the ImageJ software.

#### HSPA5/BiP protein intracellular expression by flow cytometry

Lineage-negative cells were labeled with PECy7-conjugated-Sca-1, APCCy7-conjugated-CD117, BV421-conjugated-CD135, APC-conjugated-CD48 and FITC-conjugated-CD150 for LT-HSC identification for 15 min at RT. After washing with DPBS, cells were resuspended in StemSpan™ SFEM medium, and treated or not with 1μM of 4-PBA 30 min before a 20 mGy or 0mGy irradiation. Thirty min after irradiation, cells underwent fixation and permeabilization using BD Cytofix/Cytoperm kit. Then cells were incubated with PE-conjugated-BIP antibody or PE-conjugated-IgG as a control for 45 min at 4°C in in BD Perm/Wash buffer containing 0.5% BSA. After washing, cells were resuspended in DPBS for FACS analysis.

#### Colony-forming unit (CFU)

On day 6 after culture, 20mGy- and 0 Gy-LT-HSC were sorted (200 cells) and treated or not with 1μM of 4-PBA for 30 min and plated in methylcellulose medium to support the development of hematopoietic colonies. Colonies were counted on day 8, with replicate plates used for each condition.

A secondary plating of 20,000 cells from primary colonies was performed and new colonies formed were counted after 7 days.

#### Cytogenetic analyses of blood B-cells and bone marrow LSK

Blood was collected from mice engrafted with either 0Gy- or 20mGy-LSK via intracardiac sampling under isoflurane anesthesia. Red blood cells were removed using NH_4_Cl solution (STEMCELL Technologies). After lysis and centrifugation, the cells were resuspended in 2% FCS and 1mM EDTA in DPBS buffer. B-cells were isolated using the EasySep™ Mouse B Cell Isolation kit according to the manufacturer’s protocol. Isolated B cells were cultured for 72h in RPMI 1640 medium (Gibco) supplemented with 20% FCS, 100 μg/ml Penicillin/streptomycin (Gibco) and 1% L-Glutamine (Gibco) and 50 μg/ml LPS (Lipopolysaccharide), 2.5 ng/ml mouse recombinant IL-4 and 55 μM 2-Mercaptoethanol for activation. Sorted LSK cells from bone marrow were cultured in StemSpan medium supplemented with cytokines.

Cells were treated for 4h with 10 μg/ml colchicine at 37°C and collected by centrifugation. The pellet was resuspended in a buffer composed of half 0.075M KCL and half FCS (1:6 dilution in water) and incubated for 15 min in a 37°C water bath. The cells were then centrifuged and resuspended in a fixative buffer (3/4 absolute ethanol and 1/4 acetic acid) for at least 2 days. were pre-washed and stored in distilled water at 4°C to cool before cell plating. Suspension of cells were placed on Thermo Scientific™ Plain Microscope Slides (#500) and let air dry. The M-FISH staining was applied according the Manufacturer’s protocol. Finally, slides were treated with DAPI/antifade for chromosome counterstaining and mounted. Image acquisition was performed using a Zeiss Axio Imager microscope driven by MetaSystems software.

#### Mitochondrial DNA deletion

Total DNA was extracted from sorted LSK using the QIAamp® DNA Micro kit. A semi-quantitative double PCR was performed. The first PCR was carried out using Phusion® DNA polymerase with the MITO P1 forward primer (5’-ACCAACAGCTACCATTACATT) and the MITO P2 reverse primer (5’-TTAGGTTGTTTAGTTCTAGT). The PCR product was then used as template for a second PCR using ReadyMix PCR REDExtract-N-Amp™ with the OuterP1 forward primer (5’-TCATTCTAGCCTCGTACCAAC) and the OuterP2 reverse primer (5’-GAGGTCTGGGTCATTTTCGTTA). The PCR product was loaded on a 1.5% agarose gel in TBE for visualization of the 304 bp band. A 12S PCR was also performed as a control for mitochondrial DNA, using the 12S forward primer (5’-ACCGCGGTCATACGATTAAC) and the 12S reverse primer (5’-CCCAGTTTGGGTCTTAGCTG). The 12S PCR product was loaded on a 2% agarose gel in TBE to visualized the 177 bp band. Gels were scanned using the Amersham™ Typhoon, and band intensity was measured with the ImageJ software.

#### ATAC-seq

ATAC-seq was carried out in 10,000 HSC isolated from bone marrow of mice transplanted with sham or 20mGy-irradiated LSK, in three biological replicates. In these samples, the percentage of HSC with ROS^high^ was increased by at least 1.5-fold compared to 0Gy HSC.

Cells were lysed in 10 mM Tris-HCl, 10 mM NaCl, 3 mM MgCl2, 0.1% Igepal and the tagmentation reaction was carried out in presence of Tn5 transposase (Illumina) and 0.2 mg/ml digitonin (Promega) for 30 min at 37 °C. Tagmented DNA was amplified with NEBNext High-Fidelity PCR Master Mix (NEB) and sequencing primers Ad1_noMX and Ad2.1-6 indexing primers (Illumina). Libraries were cleaned and size-selected using Spriselect beads (Beckman) and sequenced on Illumina Hiseq 4000. Paired-end 100 bp reads were mapped to the mm10 genome assembly using bowtie2 v2.3.4.3. Peak calling was performed using Macs2 v2.2.4 and differentially accessible regions were identified by DESeq2.

#### Methylome

Four months after primary transplantation of 20mGy- or 0 Gy-LSK treated or not with 1μM of 4-PBA in conditioned mice, bone marrow was harvested for donor HSC sorting. DNA was extracted according to the manufacturer’s protocol (QIAamp® DNA Micro Kit #56304, Qiagen) and sent to INTEGRAGEN for methylome analysis.

Quality of reads was assessed for each sample using FastQC (http://www.bioinformatics.babraham.ac.uk/projects/fastqc/). We used BS-Seeker2^71^ to map RRBS data to the mouse mm10 genome and retrieve the number of methylated and unmethylated cytosines at each covered CpG site. Methylation rates were then integrated across CpG island (CGI)-based and gene-based features. CGI-based features were defined as follows: CpG islands (from UCSC database mm10), shores (2 kb on each side of the island) and shelves (2 kb on each side of the shores). DNA methylation outside CpG islands was analyzed by grouping CpG sites not located in CGI-based features every 100kb window. Gene-based features were defined based on Gencode vM24 genes. We calculated for each gene the methylation rate across the promoter region (TSS +/- 500bp) and the gene body. For differential methylation analysis, we compared methylation rates across all CGI-based and gene-based features (covered by at least 50 reads). Q-values were computed by comparing the number of methylated and unmethylated reads in each condition using a logistic regression and the SLIM method for pvalue adjustment, as implemented in the methylKit package. We also calculated the methylation rate difference (delta) between each pair of test and reference sample. We considered as significantly hypermethylated (resp. hypomethylated) every region with a q-value <0.05, a difference between mean methylation levels in test and reference groups >0.05 (resp. <-0.05), and a methylation delta >0.05 (resp. <-0.05) in at least 80% of test-reference pairs.

### Quantification and statistical analysis

All statistical analyses were performed using Prism 10 (GraphPad Software) and statistic tests are specifically indicated in the legends of the figures with the number of independent biological replicates (n).
