# Supplementary material for: Heritable ER stress impairs mitochondrial metabolism and maintenance of hematopoietic stem cells after low-dose irradiation
Source: iScience. 2026 Jan 19;29(2):114738. doi: 10.1016/j.isci.2026.114738 (PMC12886522; doi:10.1016/j.isci.2026.114738)
Supplement: Document S1. Figures S1–S6 and Table S1 [file mmc1.pdf]

iScience, Volume *29*

Supplemental information

Heritable ER stress impairs mitochondrial metabolism and maintenance of hematopoietic stem cells after low-dose irradiation

Stephanie G. Moreno, Federica Ferri, Daniel Lewandowski, Vilma Barroca, Saiyiramii Devanand, Nathalie Dechamps, Paul-Henri Romeo, and Nathalie Gault

Figure S1

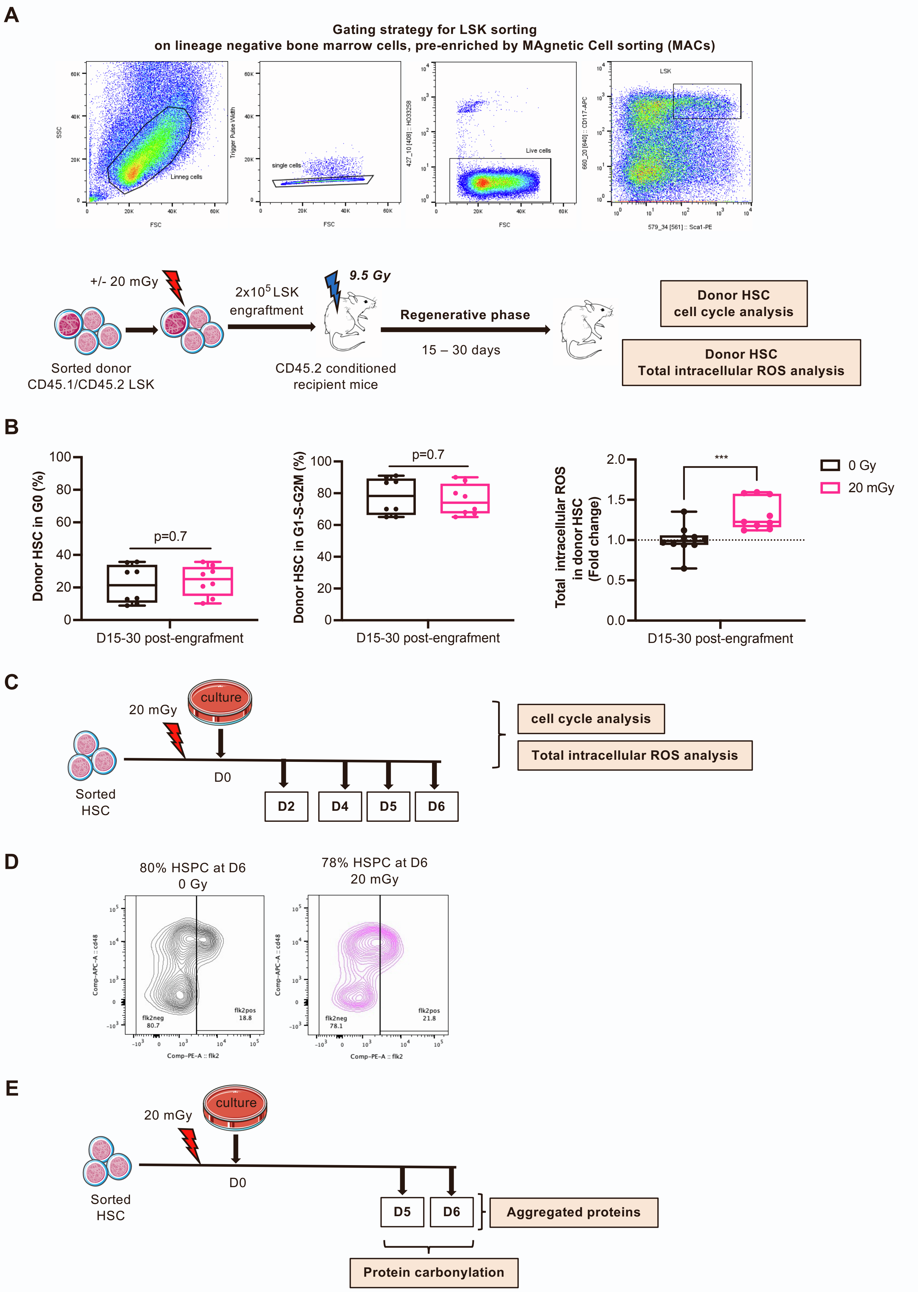

A
Gating strategy for LSK sorting
on lineage negative bone marrow cells, pre-enriched by MAgnetic Cell sorting (MACs)
SSC
FSC
Linneg cells
Trigger Pulse Width
single cells
427_10 [408] :: HO33258
Live cells
660_20 [640] :: CD117-APC
LSK
579_34 [561] :: Sca1-PE
+/- 20 mGy
Sorted donor
CD45.1/CD45.2 LSK
2x10^5 LSK
engraftment
9.5 Gy
CD45.2 conditioned
recipient mice
Regenerative phase
15 – 30 days
Donor HSC
cell cycle analysis
Donor HSC
Total intracellular ROS analysis
B
Donor HSC in G0 (%)
p=0.7
D15-30 post-engrafment
Donor HSC in G1-S-G2M (%)
p=0.7
D15-30 post-engrafment
Total intracellular ROS
in donor HSC
(Fold change)
***
D15-30 post-engrafment
0 Gy
20 mGy
C
20 mGy
culture
Sorted
HSC
D0
D2
D4
D5
D6
cell cycle analysis
Total intracellular ROS analysis
D
80% HSPC at D6
0 Gy
78% HSPC at D6
20 mGy
Comp-APC-A :: cd48
Comp-PE-A :: flk2
flk2neg
80.7
flk2pos
18.8
flk2neg
78.1
flk2pos
21.8
E
20 mGy
culture
Sorted
HSC
D0
D5
D6
Aggregated proteins
Protein carbonylation

Figure S2

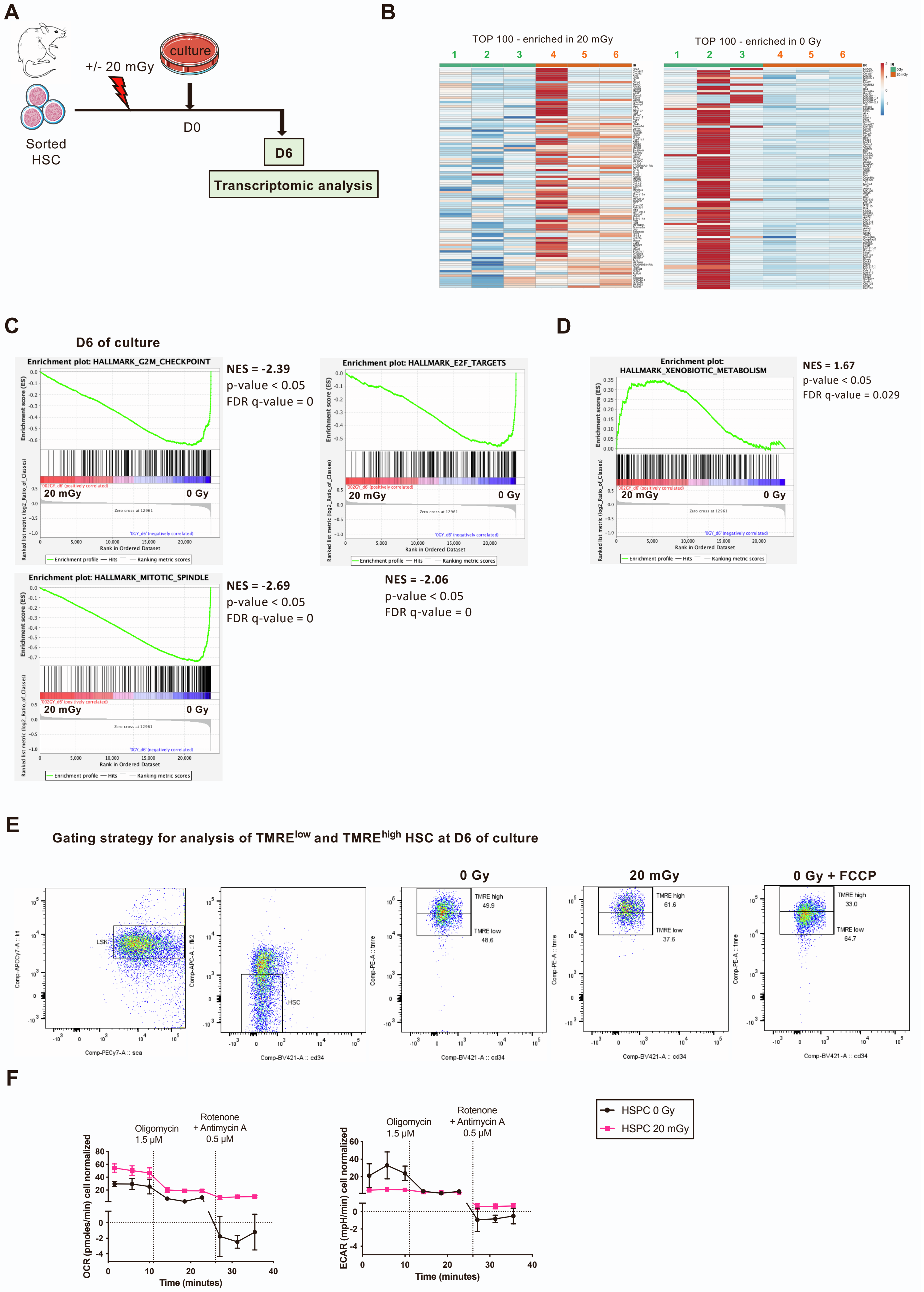

A
Sorted HSC
+/- 20 mGy
culture
D0
D6
Transcriptomic analysis
B
TOP 100 - enriched in 20 mGy
TOP 100 - enriched in 0 Gy
1 2 3 4 5 6
C
D6 of culture
Enrichment plot: HALLMARK_G2M_CHECKPOINT
NES = -2.39
p-value < 0.05
FDR q-value = 0
Enrichment plot: HALLMARK_E2F_TARGETS
NES = -2.06
p-value < 0.05
FDR q-value = 0
Enrichment plot: HALLMARK_MITOTIC_SPINDLE
NES = -2.69
p-value < 0.05
FDR q-value = 0
20 mGy
0 Gy
Enrichment score (ES)
Ranked list metric (log2_Ratio_of_Classes)
Rank in Ordered Dataset
Zero cross at 12961
Enrichment profile
Hits
Ranking metric scores
D
Enrichment plot: HALLMARK_XENOBIOTIC_METABOLISM
NES = 1.67
p-value < 0.05
FDR q-value = 0.029
E
Gating strategy for analysis of TMRE^low and TMRE^high HSC at D6 of culture
LSK
HSC
0 Gy
TMRE high 49.9
TMRE low 48.6
20 mGy
TMRE high 61.6
TMRE low 37.6
0 Gy + FCCP
TMRE high 33.0
TMRE low 64.7
Comp-APCCy7-A :: kit
Comp-PECy7-A :: sca
Comp-APC-A :: flk2
Comp-BV421-A :: cd34
Comp-PE-A :: tmre
F
Oligomycin 1.5 µM
Rotenone + Antimycin A 0.5 µM
OCR (pmoles/min) cell normalized
ECAR (mpH/min) cell normalized
Time (minutes)
HSPC 0 Gy
HSPC 20 mGy

Figure S3

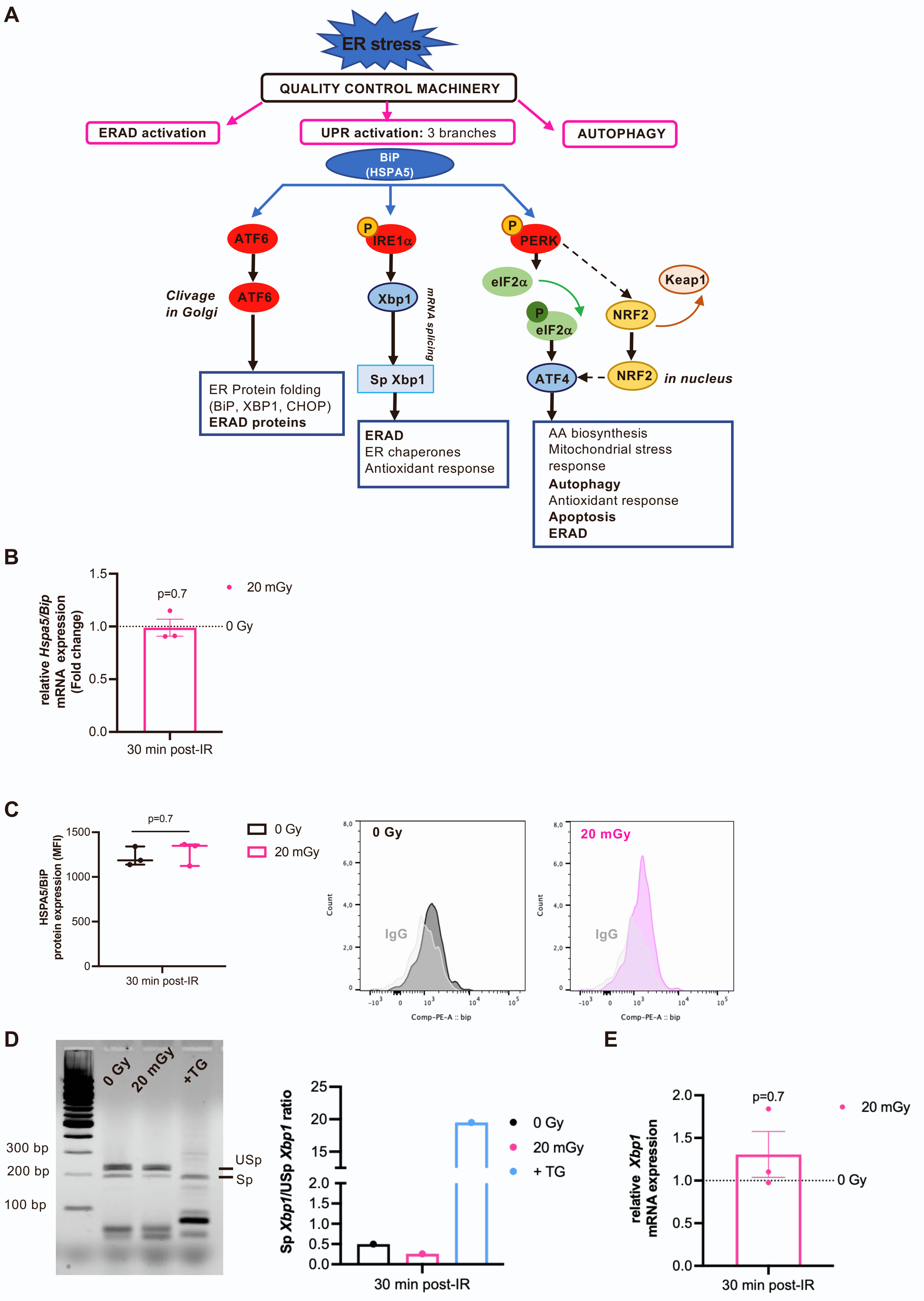

A
ER stress
QUALITY CONTROL MACHINERY
ERAD activation
UPR activation: 3 branches
AUTOPHAGY
BiP
(HSPA5)
ATF6
P
IRE1α
P
PERK
Clivage
in Golgi
ATF6
Xbp1
mRNA splicing
eIF2α
Keap1
P
eIF2α
NRF2
Sp Xbp1
ATF4
NRF2
in nucleus
ER Protein folding
(BiP, XBP1, CHOP)
ERAD proteins
ERAD
ER chaperones
Antioxidant response
AA biosynthesis
Mitochondrial stress
response
Autophagy
Antioxidant response
Apoptosis
ERAD
B
relative Hspa5/Bip
mRNA expression
(Fold change)
1.5
1.0
0.5
0.0
p=0.7
20 mGy
0 Gy
30 min post-IR
C
HSPA5/BiP
protein expression (MFI)
1500
1000
500
0
p=0.7
0 Gy
20 mGy
30 min post-IR
0 Gy
IgG
Count
Comp-PE-A :: bip
20 mGy
IgG
Count
Comp-PE-A :: bip
D
0 Gy
20 mGy
+TG
300 bp
200 bp
100 bp
USp
Sp
Sp Xbp1/USp Xbp1 ratio
25
20
15
2.0
1.5
1.0
0.5
0.0
0 Gy
20 mGy
+ TG
30 min post-IR
E
relative Xbp1
mRNA expression
2.0
1.5
1.0
0.5
0.0
p=0.7
20 mGy
0 Gy
30 min post-IR

Figure S4

A

FACS-based gating strategy for sorting Lin^{neg} Sca-1^{+} c-Kit^{+} Flk2^{neg} CD48^{neg} cells (HSC) at D6 of culture for IRE1α-Xbp1 analysis

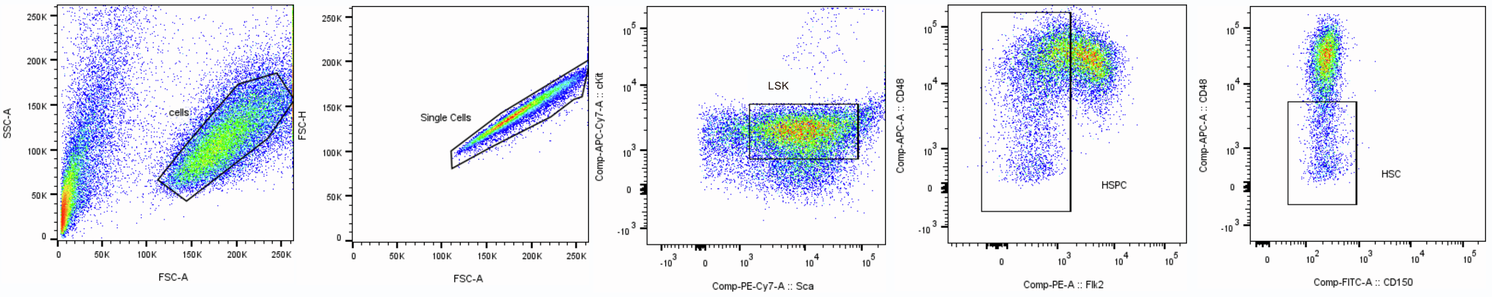

B

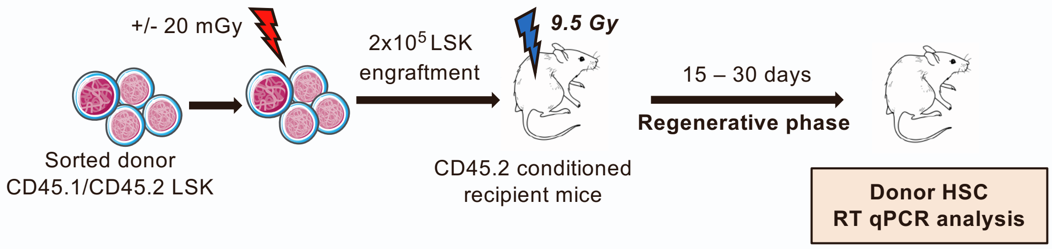

Figure S5

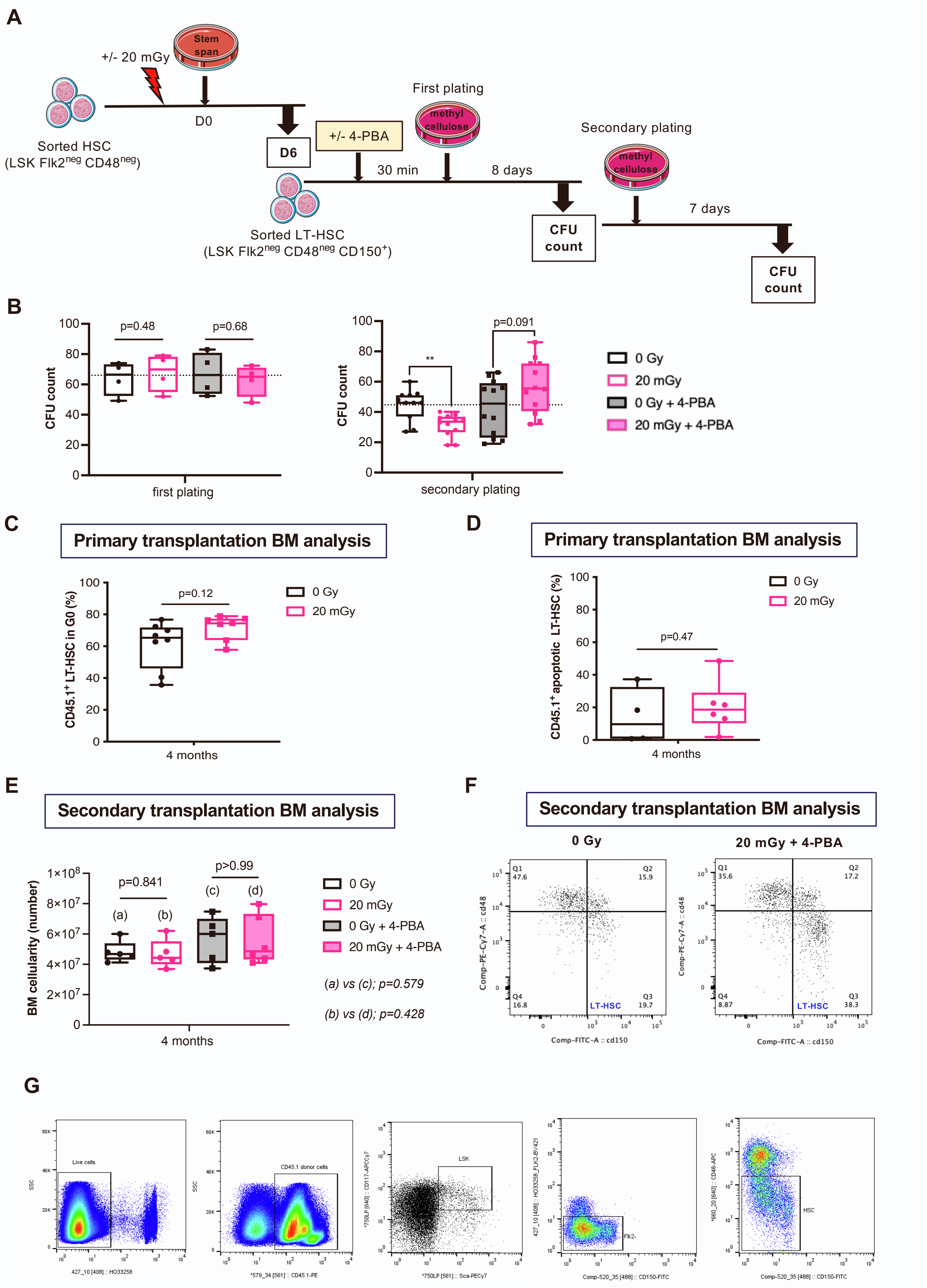
A
Stem span
+/- 20 mGy
D0
Sorted HSC
(LSK Flk2neg CD48neg)
D6
+/- 4-PBA
First plating
methyl cellulose
30 min
8 days
Sorted LT-HSC
(LSK Flk2neg CD48neg CD150+)
Secondary plating
methyl cellulose
CFU count
7 days
CFU count
B
CFU count
p=0.48
p=0.68
first plating
p=0.091
**
secondary plating
0 Gy
20 mGy
0 Gy + 4-PBA
20 mGy + 4-PBA
C
Primary transplantation BM analysis
CD45.1+ LT-HSC in G0 (%)
p=0.12
4 months
0 Gy
20 mGy
D
Primary transplantation BM analysis
CD45.1+ apoptotic LT-HSC (%)
p=0.47
4 months
0 Gy
20 mGy
E
Secondary transplantation BM analysis
BM cellularity (number)
p=0.841
p>0.99
(a)
(b)
(c)
(d)
4 months
0 Gy
20 mGy
0 Gy + 4-PBA
20 mGy + 4-PBA
(a) vs (c); p=0.579
(b) vs (d); p=0.428
F
Secondary transplantation BM analysis
0 Gy
20 mGy + 4-PBA
Comp-PE-Cy7-A :: cd48
Comp-FITC-A :: cd150
Q1 47.6
Q2 15.9
Q4 16.8
Q3 19.7
LT-HSC
Q1 35.6
Q2 17.2
Q4 8.87
Q3 38.3
G
Live cells
CD45.1 donor cells
LSK
Flk2-
HSC

Figure S6

A

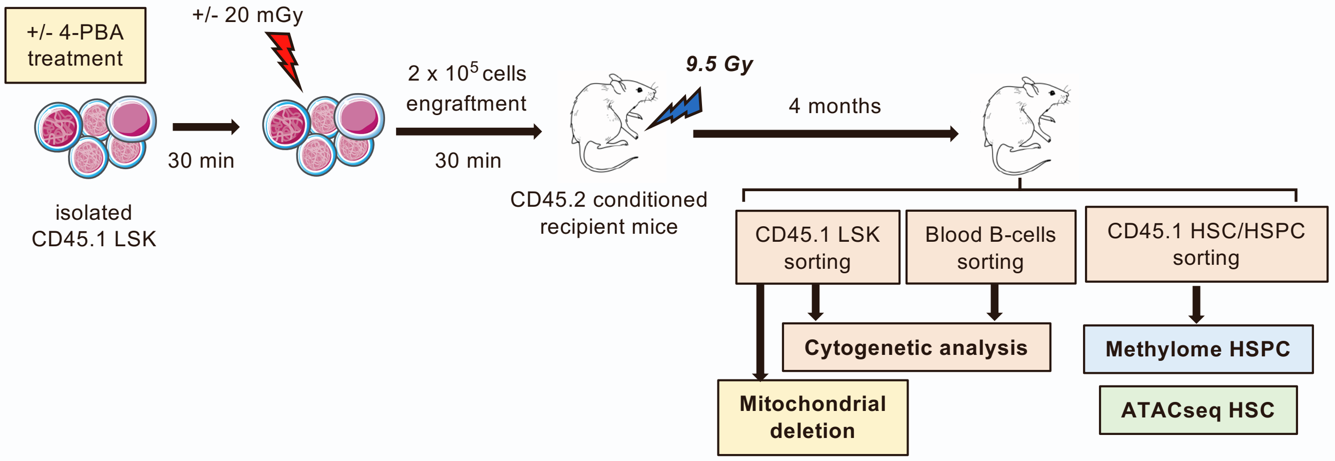

B

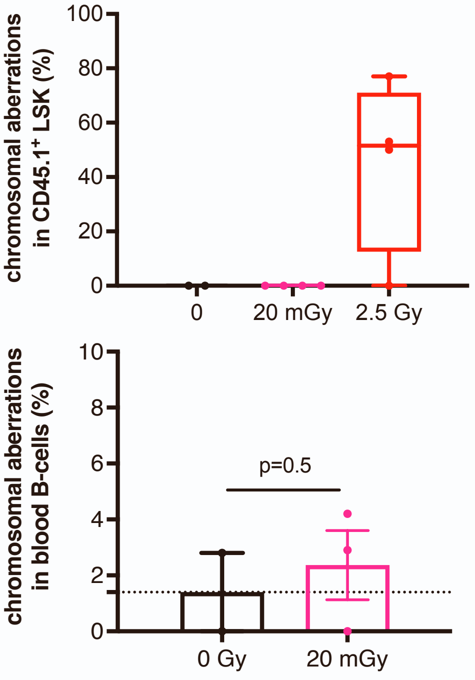

C

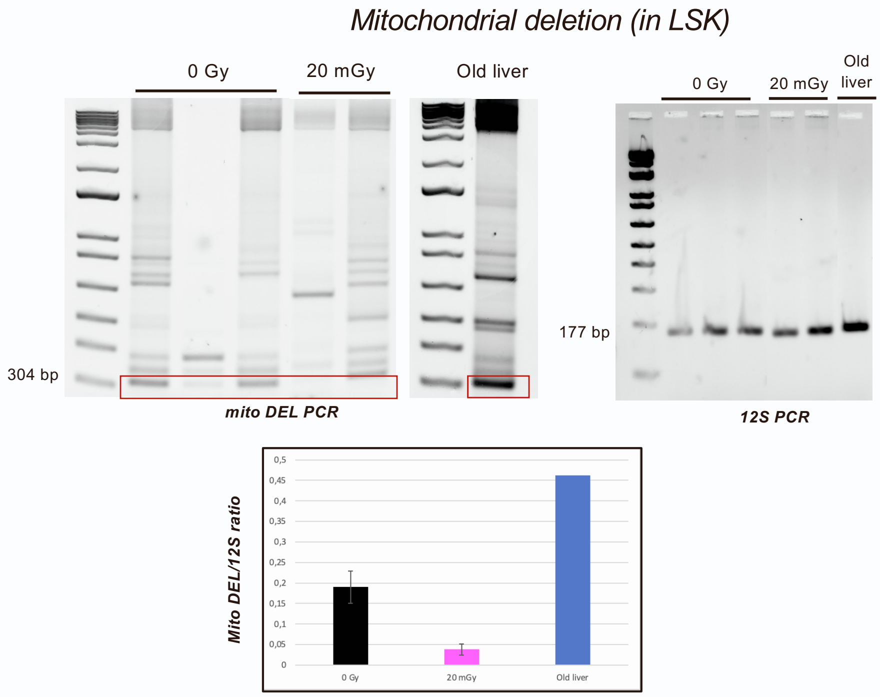

D

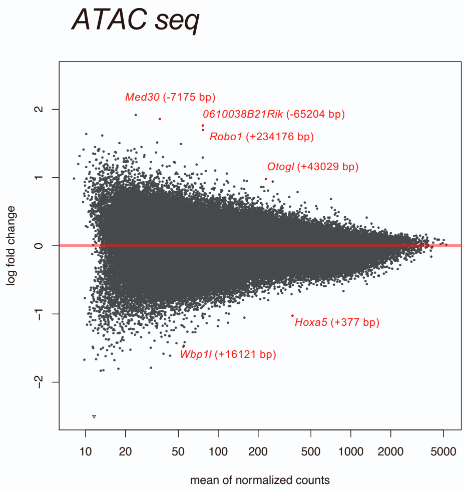

Figure S1: Following transplantation, HSC resume proliferation leading to increased ROS levels during the regeneration phase

(A) Top, FACS gating strategy for LSK sorting before transplantation. **Bottom,** Experimental design for transplantation experiments. 20,000 CD45.1+/CD45.2+ sorted LSK irradiated at 20mGy or sham-irradiated were immediately transplanted into CD45.2+ conditioned recipient mice (9.5 Gy-TBI). Cell cycle and total intracellular ROS were analyzed in donor HSC from bone marrow of recipient mice 15-30 days post-engraftment. **(B) left and middle**, Cell cycle analysis in donor HSC using Ho and Ki-67 staining at 15-30 days post-engraftment. Graphs represent the percentage of cells in G0 and in the G1-S-G2-M phase of the cell cycle. n= 8 transplanted mice per condition. **Right**, intracellular total ROS levels in donor 20mGy-HSC relative to 0Gy at 15-30 days post-engraftment. n= 8 transplanted mice per condition. Data are represented with min to max box-and-whisker. Statistical significance was assessed using a Wilcoxon-Mann-Whitney test. *** $p \leq 0.001$ **(C)** Experimental scheme showing *in vitro* culture of HSC, related to **Figure 1A** and **1B**. Sorted HSC were 20mGy irradiated or not and cultured for a maximum of 6 days. Cell cycle and total intracellular ROS analysis were performed at indicated times. **(D)** Flow cytometry analysis on 20mGy- and 0Gy-HSPC at day 6 of culture. FACS plots show the percentage of $FLK2^{neg}$ $CD48^{neg/+}$ withing the LSK population after 6 days of culture. The $CD48^{neg/+}$ gate corresponds to HSPC which include the $CD48^{neg}$ HSC subset. Number in the gate represents the percentage of HSPC relative to the parent population. **(E)** Experimental scheme related to **Figure 1C** and **1D** showing *in vitro* culture of 0Gy-HSC and 20mGyHSC for 6 days and analysis of protein carbonylation and aggregated proteins.

Figure S2: GSEA analysis between 20mGy-HSC and 0 Gy-HSC at D6 of culture

(A) Experimental scheme showing *in vitro* culture of 20mGy-HSC and 0Gy-HSC for 6 days. HSC are then sorted for transcriptomic analysis. (**B**) Heat maps of normalized expression levels of the most changed genes (TOP 100) for 20mGy (left) and 0Gy HSC (right), as determined by the GSEA ranking. Data are centered and scaled by row. The columns display the data for each of the 3 replicates. **(C)** Gene set enrichment analysis (GSEA) showing pathways involved in genome maintenance that are negatively correlated in 20mGy-HSC compared to 0Gy-HSC at day 6 of culture. **(D)** GSEA showing a significant signature for radiation-altered xenobiotic metabolism enriched in 20mGy-HSC compared to 0Gy-HSC at day 6 of culture. FDR, false discovery rate; NES, normalized enrichment score; n= 3 independent culture experiments / per condition. (**E**) FACS gating strategy for TMRE analysis in HSC at day 6 of culture. **(F)** Representative graphs showing time-course traces of normalized OCR (left) and ECAR (right), following subsequent injections of oligomycin and rotenone /antimycin A in 0 Gy versus 20mGy -enriched HSPC at day 6 of culture.

Figure S3: The ER stress occurs immediately in HSC irradiated at 20mGy without triggering the UPR pathway

(A) Protein quality control machineries of the ER consist for three axes: ER-associated degradation (ERAD), activation of the unfolded protein response (UPR) and autophagy. The UPR consists of three ER-resident transmembrane stress sensors, inositol-requiring protein 1 (IRE1); activating transcription

factor 6 (ATF6) and protein kinase RNA (PKR)-like ER kinase (PERK). Under ER stress, these UPR sensors, which are kept inactive by the chaperone-binding immunoglobulin protein (HSPA5/BiP), initiate the UPR by dissociating from BiP. The UPR facilitates the transcription of subsets of genes that restore cellular protein homeostasis and promote cell survival or apoptosis under prolonged irreversible ER stress. **(B)** *Hspa5* mRNA levels 30 min post- irradiation in 20mGy-HSC relative to 0Gy-HSC. n=3 independent experiments in duplicate. **(C) Left**, Expression of HSPA5/BiP protein 30 min post-irradiation in 0Gy and 20mGy-HSC. n=3 independent experiments. **Right**, representative flow cytometry histograms of HSPA5/BiP expression in 0Gy (dark grey solid line) and 20mGy-HSC (pink solid line). Corresponding PE-conjugated IgG (light grey line) serve as an isotype-matched control. **(D) left**, *Xbp1* mRNA Splicing 30 min post-irradiation in 0Gy and 20mGy-HSC. Treatment of HSC with thapsigargin (TG) serves as positive control. **Right**, Data are presented as the ratio of SpXbp1 to USpXbp1 mRNA. **(E)** Total *Xbp1* mRNA levels 30 min post-irradiation in 20mGy-HSC relative to 0Gy-HSC. n=3 independent cell cultures in duplicate. Data are represented with mean ± SEM or min to max box-and-whisker. Statistical significance was assessed using a Wilcoxon-Mann-Whitney (B, C, E).

Figure S4: Experimental design for transplantation experiments

(A) FACS gating strategy for HSPC or HSC sorting after day 6 of culture **(B)** 20,000 sorted CD45.1+/CD45.2+ LSK irradiated or not at 20mGy were immediately transplanted into CD45.2+ conditioned recipient mice. Donor HSC were isolated from bone marrow of recipient mice 15-30 days after transplantation and analyzed for mRNA levels for Hspa5/BiP, Atf4 and ERAD genes by RT-qPCR.

Figure S5: Functionality of 20mGy-HSC *in vitro* and *in vivo*

(A) Experimental design for *in vitro* methylcellulose Colony Formation Unit (CFU) assay. HSC were 20mGy or sham-irradiated and cultured for 6 days. HSC were then FACS sorted, treated or not with 4-PBA and cultured in the M3434 methylcellulose medium that supports the growth of primitive erythroid progenitors and myeloid progenitors. Colonies, defined as a group of > 50 cells, were counted after 8 days in culture. A secondary CFU assay was performed by replating 20,000 cells obtained by harvesting complete primary colonies. Secondary colonies were counted after 7 days in culture. **(B) Left**, Graph shows the number of primary CFU normalized to the number of seeded HSC. **Right**, Graph shows the number of secondary CFU for each condition based on 20,000 cells seeded from primary colonies. n=2-3 independent experiments in duplicate or triplicate. **(C)** Cell cycle analysis of donor CD45.1+ LT-HSC from the bone marrow of the primary recipient mice 4 months after transplantation. The graph represents the percentage of donor CD45.1+ LT-HSC in the G0 phase (Ho^{neg}, Ki-67^{neg}) of the cell cycle. n= 7-8 independent mice. **(D)** Apoptosis measurement, via annexin V/Ho analysis of donor CD45.1+ LT-HSC from the bone marrow of the primary recipient mice 4 months after transplantation. n=4-6 independent mice. **(E)** Bone marrow cellularity of the secondary recipient mice 4 months after transplantation. Graph represents median cell counts. **(F)** Representative dot plots of LT-HSC from secondary recipient mice transplanted with bone marrow from primary recipient mice transplanted with 0Gy-LSK (left) and 20mGy-LSK treated with 4-PBA (right). FACS plots show gating for CD48^{neg} CD150^{+} of LSK FLK2^{neg} defined as LT-HSC. **(G)** FACS gating strategy for donor CD45.1^{+} HSC analysis 4 months after transplantation.

All data are represented with min to max box-and-whisker. Statistical significance was assessed using a Wilcoxon-Mann-Whitney test (B, C, D, E). ** $p \leq 0.01$

Figure S6: Studies of long-term effects of 20mGy irradiation in transplanted HSC.

(A) Experimental design for transplantation experiments. 20,000 CD45.1+ LSK pre-treated or not with 4-PBA and irradiated or not at 20mGy were transplanted in CD45.2 conditioned recipient mice (9.5 Gy-TBI). Donor CD45.1+ cells were isolated from primary mice 4 months after transplantation. CD45.1+ LSK from bone marrow and B-cells from blood were isolated for cytogenetic analysis. CD45.1+ LSK were isolated for mitochondrial deletion. CD45.1+ HSC were isolated for ATACseq analysis and CD45.1+ HSPC were isolated for methylome analysis. A minimum of 3 mice per condition were used for each analysis. **(B)** Graphs represent the percentage of LSK from bone marrow (top) and the percentage of B-cell from blood (bottom) with chromosome aberrations. A dose of 2.5 Gy was used as positive control. **(C)** DNA analysis for mitochondrial deletion (**left**) and 12S ribosomal DNA (right). DNA extracted from the liver of an old mouse is used as a positive control for mitochondrial deletion (**middle**). **Lower**, Graph represents the ratio of intensities of DNA mitochondrial deletion to 12S ribosomal DNA. **(D)** Quantitative analysis of chromatin accessibility by ATAC-seq. MA-plot shows the mean versus the ratio (logFold change) of normalized read counts in 20mGy-HSC versus 0Gy-HSC. Red dots indicate differentially accessible regions in 20mGy-HSC versus 0Gy-HSC cells, that are annotated to the nearest TSS. Data are represented with mean ± SEM or min to max box-and-whisker.

Table S1: List of murine qRT-PCR primers

Genes	Forward primers	Reverse primers
Hspa5	5'- TGTCTTCTCAGCATCAAGCAAGG	5'- CCAACACTTTCTGGACAGGCTT
Total XBP1	5'- AAACAGAGTAGCAGCGCAGACTGC	5'- TCCTTCTGGGTAGACCTCTGGGAG
ATF4	5'- AACCTCATGGGTTCTCCAGCGA	5'- CTCCAACATCCAATCTGTCCCG
Asns	5'- TTACCTGTCTCTGCCGCCAGAT	5'- CACTGAAGGCTTCTTTGGGTCG
Aldh18a1	5'- CGTCATCACAGACATCGTGGAG	5'- GGCTCTAAGGTAGCCAGCATTC
Herpud1	5'- CCTCCAAAATGCCAGAAACCAGC	5'- GCCGTAAACCATCACTTGAGGAG
Sel1L	5'- GGAAGTGACATCGTACCTCAGAG	5'- CTTGAACGCCTCTTCCGTAGAG
VCP	5'- CTGGCAGATGATGTGGACTTGG	5'- CAGAGCAGCCTCTGAACATAGG
b-Actin	5'- AAGGCCAACCGTGAAAAGAT	5'- GTGGTACGACCAGAGGCATAC
GAPDH	5'- CAATGTGTCCGTCGTGGATCT	5'- TTGAAGTCGCAGGAGACAACC
